# Impact of placental mTOR deficiency on peripheral insulin signaling in adult mice offspring

**DOI:** 10.1530/JME-23-0035

**Published:** 2023-10-18

**Authors:** Megan Beetch, Brian Akhaphong, Alicia Wong, Briana Clifton, Seokwon Jo, Ramkumar Mohan, Juan E Abrahante Llorens, Emilyn U Alejandro

**Affiliations:** 1 Department of Integrative Biology and Physiology, University of Minnesota Medical School, Minneapolis, Minnesota, USA; 2 University of Minnesota Informatics Institute (UMII), Minneapolis, Minnesota, USA

**Keywords:** mTOR, insulin sensitivity, diabetes, placenta, fetal programming, MUP1

## Abstract

Suboptimal *in utero* environments such as poor maternal nutrition and gestational diabetes can impact fetal birth weight and the metabolic health trajectory of the adult offspring. Fetal growth is associated with alterations in placental mechanistic target of rapamycin (mTOR) signaling; it is reduced in fetal growth restriction and increased in fetal overgrowth. We previously reported that when metabolically challenged by a high-fat diet, placental mTORKO (mTORKO^pl^) adult female offspring develop obesity and insulin resistance, whereas placental TSC2KO (TSC2KO^pl^) female offspring are protected from diet-induced obesity and maintain proper glucose homeostasis. In the present study, we sought to investigate whether reducing or increasing placental mTOR signaling *in utero* alters the programming of adult offspring metabolic tissues preceding a metabolic challenge. Adult male and female mTORKO^pl^, TSC2KO^pl^, and respective controls on a normal chow diet were subjected to an acute intraperitoneal insulin injection. Upon insulin stimulation, insulin signaling via phosphorylation of Akt and nutrient sensing via phosphorylation of mTOR target ribosomal S6 were evaluated in the offspring liver, white adipose tissue, and skeletal muscle. Among tested tissues, we observed significant changes only in the liver signaling. In the male mTORKO^pl^ adult offspring liver, insulin-stimulated phospho-Akt was enhanced compared to littermate controls. Basal phospho-S6 level was increased in the mTORKO^pl^ female offspring liver compared to littermate controls and did not increase further in response to insulin. RNA sequencing of offspring liver identified placental mTORC1 programming-mediated differentially expressed genes. The expression of major urinary protein 1 (*Mup1*) was differentially altered in female mTORKO^pl^ and TSC2KO^pl^ offspring livers and we show that MUP1 level is dependent on overnutrition and fasting status. In summary, deletion of placental mTOR nutrient sensing *in utero* programs hepatic response to insulin action in a sexually dimorphic manner. Additionally, we highlight a possible role for hepatic and circulating MUP1 in glucose homeostasis that warrants further investigation.

## Introduction

The Developmental Origins of Health and Disease (DOHaD) hypothesis states that environmental perturbations and nutritional insults during the earliest stages of human development impact risk for adult noncommunicable disease. A prominent determinant of adverse long-term health is fetal growth restriction (FGR), primarily caused by placental dysfunction and identified in 3–7% of all pregnancies and more than a quarter of live births in low- and middle-income countries (Romo *et al.* 2009, Black 2015, Sharma *et al.* 2016). Common pregnancy complications like maternal malnutrition and preeclampsia result in placental insufficiency and FGR (Beetch & Alejandro 2021).

The placenta, an endocrine organ formed early in pregnancy to support fetal growth, responds to maternal nutrient availability and fetal demand to orchestrate the allocation of nutrients to meet energy and nutritional needs. Placental signaling pathways regulate fetal growth by sensing and integrating nutrient signals. The mechanistic target of rapamycin (mTOR) is a nutrient sensor kinase that is reported to be downregulated in the human FGR placentae and upregulated in the placentae of fetal overgrowth (Roos *et al.* 2009, Jansson *et al.* 2013, Dimasuay *et al.* 2016). The mTOR signaling pathway consists of mTOR complex 1 (mTORC1) and mTOR complex 2 (mTORC2), which are activated in response to nutrient and growth factor signals, and ultimately promote cellular growth. Using murine models of direct mTOR manipulation in the placental trophoblast cells (loss or gain of mTOR activity by deletion of mTOR or TSC2, a negative regulator of mTORC1, respectively), we demonstrated a role for placental mTORC1 signaling in modulating birth weight and adult offspring susceptibility to obesity, insulin resistance, and type 2 diabetes (T2D) (Akhaphong *et al.* 2021). Specifically, deleting placental mTOR resulted in reduced placental and fetal weights, indicating that loss of placental mTOR signaling is sufficient to alter birthweight. Interestingly, reductions in placental and fetal weight were specific to females, despite comparable baseline expression of placental mTOR when directly comparing sexes (Cowell *et al.* 2020).

T2D is a complex disease characterized by insufficient pancreatic beta-cell function and increased peripheral insulin resistance (DeFronzo 2004, Eizirik *et al.* 2020). Glucose homeostasis is tightly regulated by a cooperative physiology of multiple tissues throughout the body, including the pancreas, liver, adipose tissue, and skeletal muscle (DeFronzo 2004). In our previous placental mTOR manipulation study that defined offspring phenotypes, we found that in female offspring, the loss of placental mTOR (mTORKO^pl^) increased susceptibility to insulin resistance and obesity under a high-fat diet (HFD) challenge, whereas enhanced placental mTORC1 activity (TSC2KO^pl^) conferred protection (Akhaphong *et al.* 2021).

The liver, adipose tissue, and skeletal muscle play vital roles in the anabolic hormone effect of insulin to maintain glucose homeostasis. Insulin signaling in peripheral tissues has classically been used as a measure of insulin sensitivity or resistance. Downstream of the insulin receptor is Akt, which is rapidly activated through IRS/PI3K in response to insulin binding to the insulin receptor (Saltiel 2021). Therefore, Akt phosphorylation is used as a proxy to measure insulin signaling. Preclinical studies have shown that impaired hepatic Akt signaling is associated with hyperinsulinemia and insulin resistance (Santoleri & Titchenell 2019). Full suppression of hepatic Akt results in severe insulin resistance and glucose intolerance (Lu *et al.* 2012, Titchenell *et al.* 2016). A relationship exists between Akt and the major nutrient sensor kinase mTOR. Full activation of Akt requires mTORC2-mediated phosphorylation of serine 473 (Sarbassov *et al.* 2005). Moreover, mouse models of maternal obesity have shown impaired Akt signaling in the liver, adipose tissue, and skeletal muscle of male offspring (Martin-Gronert *et al.* 2010, 2016, Latouche *et al.* 2014). We aimed to assess insulin signaling and mTOR nutrient sensing in the liver, adipose tissue, and skeletal muscle of adult mTORKO^pl^ and TSC2KO^pl^ offspring in response to acute insulin treatment.

We previously reported an increase in insulin resistance via insulin tolerance test in the mTORKO^pl^ female adult offspring under metabolic stress by HFD-induced obesity (Akhaphong *et al.* 2021). In the present study, we hypothesized that Akt signaling and mTOR signaling are altered in metabolic peripheral tissues from mTORKO^pl^ adult offspring before metabolic stress onset as a result of altered fetal programming of these tissues *in utero*. Here, we found that hepatic Akt signaling and mTOR signaling are enhanced in mTORKO^pl^ adult offspring in a sexually dimorphic manner. Furthermore, alterations in offspring hepatic gene expression suggest that *Mup1*, a gene previously implicated in the regulation of glucose homeostasis, may also be affected by placental mTOR manipulation. Our data demonstrate direct evidence of differential fetal programming of offspring metabolic tissues following placental mTOR alteration *in utero*.

## Materials and methods

### Mouse model of loss or gain of placental mTOR function

Murine models were generated with mTOR signaling loss- or gain-of-function in the placenta. A placental trophoblast-specific Cre recombinase transgene was used and driven by the Cyp19 promoter (Wenzel & Leone 2007) with *loxP*-flanked sites in either the *mTOR* or *TSC2* gene (*mTOR^fl^^/^^fl^* or *TSC2^fl/^^fl^*). The *mTOR^fl^^/^^fl^* and *TSC2^fl/^^fl^* were purchased from The Jackson Laboratory (mTOR flox: B6.129S4-Mtor*^tm1.2Koz^*IJ, strain # 011009; TSC2 flox: *Tsc2^tm1.Mjg^I*J, stock # 027458). The Cyp19-Cre mice were donated by G. Leone from The Ohio State University. We and others have demonstrated that Cyp19 is exclusively expressed in the labyrinth zone of the placenta and not in the fetus (López-Tello *et al.* 2019, Akhaphong *et al.* 2021). Offspring used in this study were from eight Cyp19-cre; mTOR dams and seven Cyp19-cre; TSC2 dams. Littermate *mTOR^fl^^/^^fl^* and *TSC2^fl/^^fl^* were used as controls in our experiments. All mice were generated on a mixed background (C57BL/6JxFVB) and group housed on a 14 h light:10 h darkness cycle with access to normal chow diet (NCD; Teklad Global 18% Protein Rodent Diet) *ad libitum*. For high-fat (60% kcal) feeding experiments, treatment began at 15 weeks of age and lasted for 12 weeks. For rigor, sex was considered as an independent variable, and data were segregated and analyzed separately. The Institutional Animal Care and Use Committee at the University of Minnesota approved all animal studies.

### Acute insulin injection *in vivo*

Following a 10-h overnight fast, a subset of mice was randomized into intraperitoneal injection of 1 U/kg insulin or an equivalent volume of saline as a control. After 7 min, mice were euthanized with CO_2_. The liver, inguinal white adipose tissue, and skeletal muscles of the calf (soleus and gastrocnemius) were flash frozen and stored at −80°C until sample preparation.

### Protein extraction and Western blotting

Approximately 25–30 mg of tissue was lysed by homogenization and sonication in RIPA buffer (Cell Signaling Technologies), 1% SDS, and protease and phosphatase inhibitor cocktails (Cell Signaling Technologies). Following a Pierce BCA assay (ThermoScientific) to quantify protein, 35–50 μg of protein lysate was resolved on 10% SDS-PAGE, transferred to PVDF membrane, blocked with 5% nonfat dry milk, and incubated with primary antibodies overnight, prior to treatment with HRP-conjugated secondary antibodies. The primary antibodies used were as follows: phospho-Akt (S473) (Cell Signaling Technologies, 4060S) at 1:1000, Akt-pan (40D4) (Cell Signaling Technologies, 2920S) at 1:500, phospho-S6 (S240/244) (Cell Signaling Technologies, 5364S) at 1:1000, S6 ribosomal protein (Cell Signaling Technologies, 2317S) at 1:1000, MUP (Santa Cruz Biotechnology, sc-166429) at 1:500, and vinculin (Cell Signaling Technologies, 13901S) at 1:1000. The blot was visualized with SuperSignal West Pico PLUS (ThermoScientific), per the manufacturer’s instructions. Densitometry analysis was performed with NIH ImageJ/FIJI software. Values are presented relative to each respective saline control group.

### RNA extraction, RT-qPCR, and RNA sequencing analysis

RNA samples for RNA sequencing were isolated from the adult liver using the RNeasy Plus Micro Kit (Qiagen), following the manufacturer’s instructions. RNA was isolated from nonfasted animals. RNA integrity (>8) was validated using an Agilent 2200 TapeStation. During sequencing, 125 bp FastQ paired-end reads were trimmed using Trimmomatic (v 0.33; Potsdam, Germany) enabled with the optional ‘-q’ option; 3 bp sliding window trimming from 3′ end requiring minimum Q30. Quality control checks on raw sequence data for each sample were performed by FastQ. Read mapping was performed via Hisat2 (v2.0.2; Dallas, TX, and Baltimore, MD, USA), using the UCSC mouse genome (mm10) as reference. Gene quantification was performed via Feature Counts for raw read counts. Differentially expressed genes (DEGs) were identified by using the edgeR (negative binomial) feature in CLC Genomics Workbench, using raw read counts. The generated list was filtered based on a minimum 2× absolute fold-change and *P* < 0.05 false discovery rate (FDR).

For RT-qPCR, the High-Capacity cDNA Reserve Transcription Kit (Applied Biosystems) was used for the cDNA conversion of mRNA. RNA from RNAseq was used for validation experiments except for *Mup1* where RNA from RNAseq and additional mTORKO^pl^ offspring liver samples were used. Quantitative PCR was performed using the Applied Biosystems Q6 machine and SYBR Green reagent. Beta-actin and 36B4 were used as reference genes. We found no differences in results using either reference gene; therefore, the RT-qPCR results presented throughout this article are the gene of interest normalized to beta-actin. RT-qPCR primer sequences can be found in Supplementary Table 1 (see section on supplementary materials given at the end of this article).

Secondary analysis of mTORKO^pl^ versus TSC2KO^pl^ offspring liver RNA sequencing comparison yielded enough DEGs to use the online tool DAVID Knowledgebase to assess Gene Ontology and KEGG pathways. Additional confirmation of altered canonical pathways and analysis of top diseases were performed using Ingenuity Pathway Analysis.

### MUP1 ELISA

MUP1 levels from nonfasting, fasting, and HFD serum were measured using ID Mouse MUP-1 ELISA (DiaPharma, Kalamazoo, MI, USA), according to kit instructions.

### AML12 murine hepatocyte cells line and treatments

AML12 murine hepatocyte-derived cells were a gift from Dr Douglas Mashek (University of Minnesota, USA). AML12 cells were cultured in DMEM/F12 medium (Gibco) containing 10% FBS, 5 μg/mL insulin, 5 μg/mL transferrin, 5 ng/mL selenium, and 40 ng/mL dexamethasone, and maintained at 37°C in a humidified environment with 5% CO_2_. Rapamycin was dissolved in DMSO prior to use. AML12 cells were treated at 15 nM or 30 nM concentrations for 24 h prior to collection. For 12-h experiments, AML12 cells were treated with 30 nM rapamycin prior to collection. Rapamycin concentrations and timing were used to inhibit mTORC1 without impacting mTORC2.

### Intraperitoneal rapamycin injection *in vivo*

Adult female mice were injected with 2 mg/kg body weight of rapamycin per day for 10 days. Nonfasting serum and liver tissue were collected on day 11, flash frozen, and stored at −80°C until sample preparation. Western blotting and MUP1 ELISA were performed as described earlier.

### Statistical analysis

Data are represented as mean ± s.e.m. Data were analyzed using an unpaired, two-tailed *t*-test, a one-way ANOVA with Tukey *post hoc* test, or a repeated measures two-way ANOVA. Analyses and data visualization were performed in GraphPad PRISM, version 8, with a significance threshold of *P* < 0.05.

## Results

### Body weight, tissue weight, blood glucose, and serum insulin levels of placental mTORKO and TSC2KO offspring on normal chow diet

To assess whether mTORKO^pl^ and TSC2KO^pl^ offspring had differences in baseline parameters compared to littermate controls, their body weight, nonfasting blood glucose, fasting blood glucose, nonfasting serum insulin, and fasting serum insulin were measured prior to insulin injection and sacrifice. Male and female mTORKO^pl^ and TSC2KO^pl^ offspring had comparable body weights to their littermate controls (Supplementary Fig. 1A and B’). Pancreas weight was not different by genotype for either sex (Supplementary Fig. 1C and D’). Our past study found that endocrine-cell mass was not altered in mTORKO^pl^ and TSC2KO^pl^ offspring compared to their littermate controls (Akhaphong *et al.* 2021). Liver weight (Supplementary Fig. 1E and F) and gross morphology (Supplementary Fig. 1G) were also not different by genotype. Nonfasting blood glucose level and nonfasting serum insulin were normal across sexes and genotypes (Supplementary Fig. 2A, B, C, and D’). Likewise, fasting blood glucose (Supplementary Fig. 2E and F’) and fasting serum insulin (Supplementary Fig. 2G and H’) were comparable between mTORKO^pl^ and TSC2KO^pl^ offspring and their respective littermate controls.

### Enhanced Akt signaling in response to insulin stimulation in the liver of male mTORKO^pl^ adult offspring

Next, we measured insulin signaling in metabolic peripheral tissues with the knowledge that, when exposed to an HFD, mTORKO^pl^ female offspring experience increased insulin resistance and obesity than controls (Akhaphong *et al.* 2021). To assess if the insulin signaling response in adulthood was altered by the *in utero* environment induced by placental mTOR loss, we treated mice with insulin acutely after 10 h of fasting and measured Akt signaling in the peripheral tissues by Western blotting. The serine/threonine kinase Akt is a major downstream target of the insulin receptor, which signals to promote cellular growth and glucose transporter translocation to the plasma membrane for glucose uptake. Thus, phosphorylated Akt (phospho-Akt) is commonly used as a proxy for insulin signaling. To understand whether the insulin signaling pathway is programmed differently upon exposure to the mTOR-manipulated placenta *in utero*, we measured phospho-Akt in response to insulin stimulation in peripheral tissues important for glucose handling and homeostasis.

**Figure 1:**
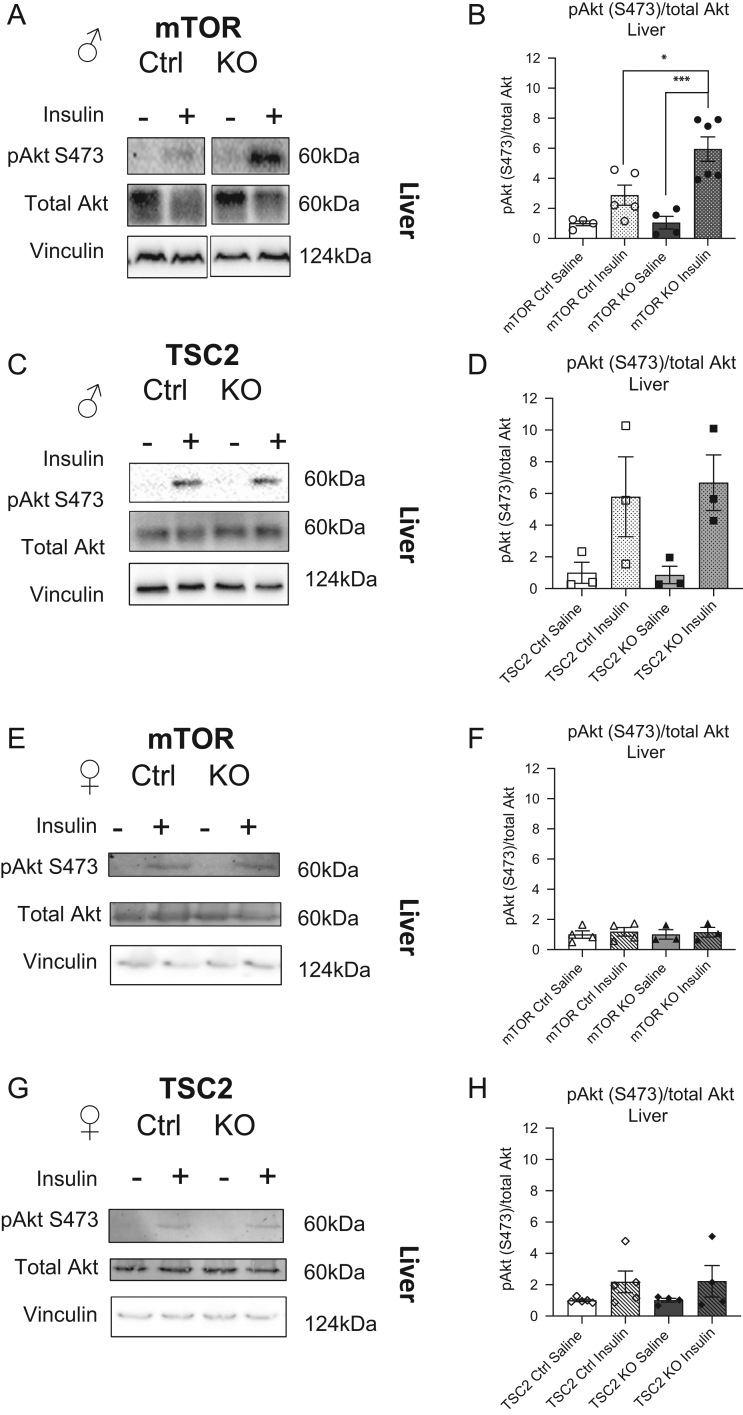
Insulin-Akt signaling in placental mTORKO^pl^ adult male and female offspring liver in response to insulin stimulation. (A) Representative Western blot of phosphorylated Akt at serine 473, total Akt, and vinculin from livers of 90-day-old mTOR control and mTORKO^pl^ male offspring injected with saline or 1 U/kg insulin. (B) Quantification of liver Western blots represented as fold-change relative to saline-treated mTOR control (*n* = 4–6). (C) Representative Western blot of phosphorylated Akt at serine 473, total Akt, and vinculin from livers of 90-day-old TSC2 control and TSC2KO^pl^ male offspring injected with saline or 1 U/kg insulin. (D) Quantification of liver Western blots represented as fold-change relative to saline-treated TSC2 control (*n* = 3). (E) Representative Western blot of phosphorylated Akt at serine 473, total Akt, and vinculin from livers of 90-day-old mTOR control and mTORKO^pl^ female offspring injected with saline or 1 U/kg insulin. (F) Quantification of liver Western blots represented as fold-change relative to saline-treated mTOR control (*n* = 3–4). (G) Representative Western blot of phosphorylated Akt at serine 473, total Akt, and vinculin from livers of 90-day-old TSC2 control and TSC2KO^pl^ female offspring injected with saline or 1 U/kg insulin. (H) Quantification of liver Western blots represented as fold-change relative to saline-treated TSC2 control (*n* = 4–5). Statistical analyses were conducted using one-way ANOVA with Tukey’s *post hoc* test, with significance *P < 0.05, ***P < 0.001.

In the male offspring liver, insulin stimulation led to higher activation of phospho-Akt in the mTORKO^pl^ (gray checked bar) offspring compared to the insulin-stimulated mTOR controls (white checked bar; Fig. 1A, quantified in Fig. 1B). Insulin stimulation increased phospho-Akt to comparable levels in the livers of TSC2 male offspring (Fig 1C, quantified in Fig 1D). We found no differences in the total Akt protein expression in mTORKO^pl^ or TSC2KO^pl^ male offspring liver compared to their littermate controls (Fig. 1A and C, quantified in Supplementary Fig. 4A and B). These results suggest differential programming of insulin signaling activity specific to the mTORKO^pl^ male offspring liver.

### No differences in Akt signaling in adipose tissue or skeletal muscle of male mTORKO^pl^ or male TSC2KO^pl^ adult offspring

Adipose tissue and skeletal muscle are important organs for energy storage and maintaining blood glucose levels. We investigated Akt signaling in the inguinal white adipose depot and the skeletal muscles of the calf (soleus and gastrocnemius) upon insulin stimulation. We found that insulin stimulation robustly increased phospho-Akt in mTORKO^pl^ and TSC2KO^pl^ adipose tissue and respective littermate controls (Supplementary Fig. 3A quantified in Supplementary Fig. 3B and C). However, there were no significant differences between controls and mTORKO^pl^ or TSC2KO^pl^ responses to insulin stimulation (Supplementary Fig. 3A quantified in Supplementary Fig. 3B and C), suggesting that programming of insulin signaling in the adipose tissue is not impacted by placental mTOR nutrient sensing in adult male mice on NCD. Interestingly, activation of phospho-Akt in response to the insulin injection was not significantly elevated in the skeletal muscle (Supplementary Fig. 3D, quantified in Supplementary Fig. 3E and F). The skeletal muscle response to insulin stimulation appears to be minimal and variable in the male offspring, perhaps due to insulin resistance observed in males at 3 months of age compared to females (Jo *et al.* 2023). Alternatively, the time course of tissue harvest at 7 min post injection may not have been enough time to optimally increase skeletal muscle insulin signaling in our model. In the male mTORKO^pl^ and TSC2KO^pl^ offspring, total Akt protein was unchanged by genotype or insulin treatment in the adipose tissue (Supplementary Fig. 3A, quantified in Supplementary Fig. 4A’ and B’) and skeletal muscle (Supplementary Fig. 3D, quantified in Supplementary Fig. 4A’’ and B’’).

### No differences in Akt signaling in female mTORKO^pl^ or TSC2KO^pl^ offspring peripheral tissues

Fetal programming can have sexually dimorphic effects in the offspring. In fact, our previously published findings indicate that deleting placental mTOR results in sexually dimorphic metabolic phenotypes (Akhaphong *et al.* 2021). Therefore, insulin-Akt signaling analysis was done in both sexes. We found that insulin stimulation did not significantly elevate phospho-Akt in the liver of mTORKO^pl^ (Fig. 1E, quantified in Fig. 1F) and TSC2KO^pl^ female offspring (Fig. 1G, quantified in Fig. 1H) compared to their respective saline-treated controls. In the inguinal white adipose depot of mTORKO^pl^ and TSC2KO^pl^ female offspring, phospho-Akt was increased upon insulin injection compared to saline-treated controls (Supplementary Fig. 3G, quantified in Supplementary Fig. 3H and I). The skeletal muscle response to insulin stimulation appears normal in the mTORKO^pl^ female offspring (Supplementary Fig. 3J, quantified in Supplementary Fig. 3K), in contrast to the blunted and variable response of the male offspring. However, TSC2KO^pl^ female offspring display only a mild increase in phospho-Akt levels in response to insulin (Supplementary Fig. 3J, quantified in Supplementary Fig. 3L). When comparing the insulin response in control and mTORKO^pl^ female offspring tissues, we found no differences in phospho-Akt in any tissue of interest. Likewise, no differences in Akt signaling were detected between control and TSC2KO^pl^ female offspring tissues in response to insulin stimulation. We did further analyses to confirm that insulin-stimulated phospho-Akt did not differ in the liver, adipose tissue, and skeletal muscle of mTORKO^pl^ and TSC2KO^pl^ female offspring compared to littermate controls by normalizing phospho-Akt to our loading control (Supplementary Fig. 5A, B, C, D, and F). Total Akt protein levels were unchanged across genotypes and in response to insulin stimulation in all female offspring tissues (Fig. 1E and G, Supplementary Fig. 3G and 3J, quantified in Supplementary Fig. 4C and D’’) Together, these data suggest the female offspring that experienced placental mTOR manipulation *in utero* do not display differences in insulin signaling, a proxy for insulin sensitivity, in the liver, adipose tissue, or skeletal muscle on NCD.

### Enhanced basal S6 signaling in the mTORKO^pl^ female offspring liver

**Figure 2:**
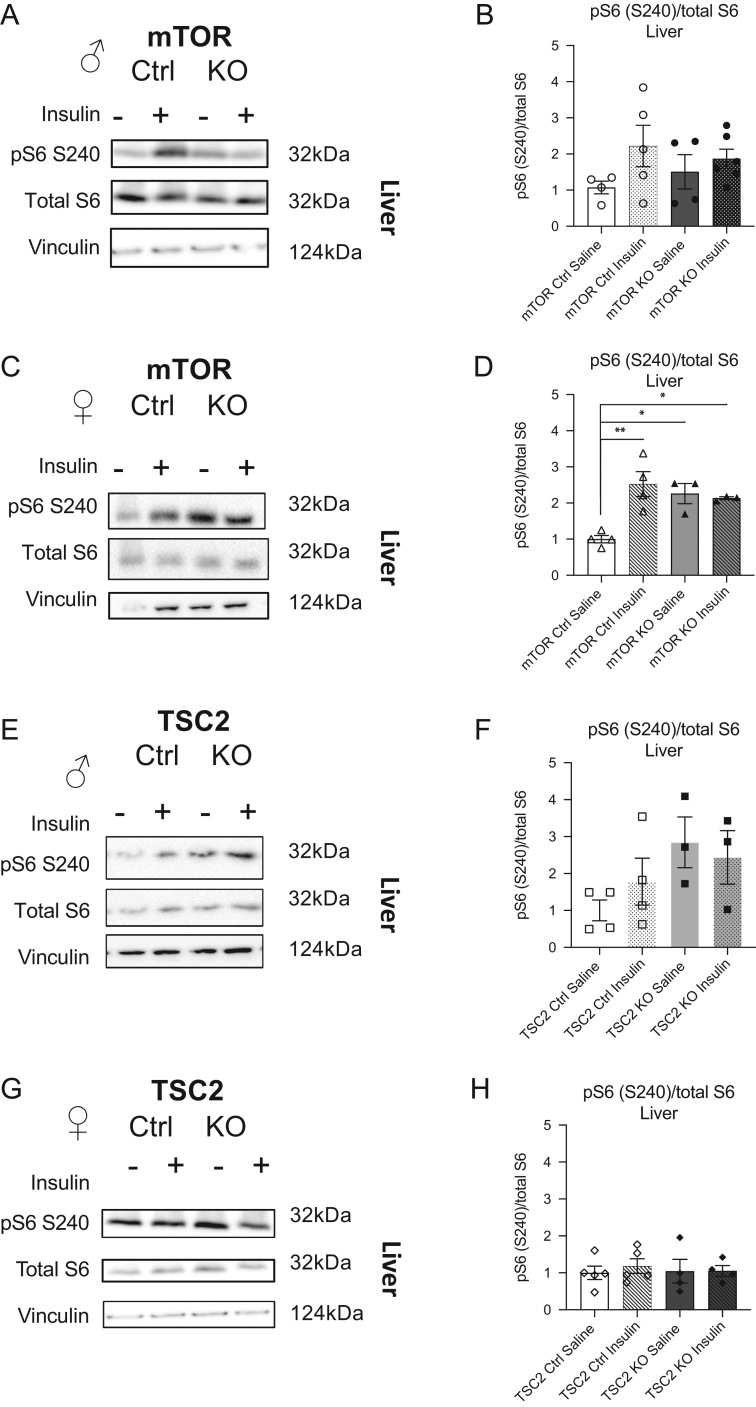
mTOR-S6 signaling is basally elevated in the placental mTORKO^pl^ female offspring liver and does not increase in response to insulin stimulation. (A) Representative Western blots of phosphorylated S6 at serine 240/244, total S6, and vinculin from livers of 90-day-old mTOR control and mTORKO^pl^ male offspring injected with saline or 1 U/kg insulin. (B) Quantification of Western blots represented as fold-change relative to saline-treated male mTOR control (*n* = 4–6). (C) Representative Western blots of phosphorylated S6 at serine 240/244, total S6, and vinculin from livers of 90-day-old mTOR control and mTORKO^pl^ female offspring injected with saline or 1 U/kg insulin. (D) Quantification of Western blots represented as fold-change relative to saline-treated female mTOR control (*n* = 3–4). (E) Representative Western blots of phosphorylated S6 at serine 240/244, total S6, and vinculin from livers of 90-day-old TSC2 control and TSC2O^pl^ male offspring injected with saline or 1 U/kg insulin. (F) Quantification of Western blots represented as fold-change relative to saline-treated male TSC2 control (*n* = 4–5). (G) Representative Western blots of phosphorylated S6 at serine 240/244, total S6, and vinculin from livers of 90-day-old TSC2 control and TSC2KO^pl^ female offspring injected with saline or 1 U/kg insulin. (H) Quantification of Western blots represented as fold-change relative to saline-treated female TSC2 control (*n* = 3). Statistical analyses were conducted using one-way ANOVA with Tukey’s *post hoc* test, with significance *P < 0.05, **P < 0.01.

To ascertain what effect placental mTOR manipulation has on nutrient sensing in offspring liver, we measured mTOR signaling by assessing the downstream phosphorylated target ribosomal protein S6 (phospho-S6). Mouse studies have indicated that phosphorylation of S6 is involved in the regulation of cell size, proliferation, and glucose homeostasis (Ruvinsky *et al.* 2005). We probed for phospho-S6 upon acute insulin stimulation and found that, in the mTORKO^pl^ male offspring liver, phospho-S6 was not significantly different compared to littermate controls (Fig. 2A, quantified in Fig. 2B). Total S6 protein levels were also unchanged (Supplementary Fig. 6A).

Insulin stimulation significantly increased phospho-S6 in the mTOR control female offspring liver (white solid bar versus white checked bar; Fig. 2C, quantified in Fig. 2D). However, in the mTORKO^pl^ female offspring liver, S6 signaling was basally elevated and did not further increase in response to insulin stimulation (gray solid bar versus gray checked bar; Supplementary Fig. 2D). Total S6 protein levels were not significantly changed in mTOR control or mTORKO^pl^ female offspring liver treated with insulin relative to their saline-treated counterparts (Supplementary Fig. 6B). TSC2KO^pl^ male (Fig. 2E, quantified in Fig. 2F) and female (Fig. 2G, quantified in Fig. 2H) offspring livers did not show significant differences in S6 signaling in response to insulin injection.

### Differentially expressed genes in the mTORKO^pl^ and TSC2KO^pl^ adult offspring livers

**Figure 3:**
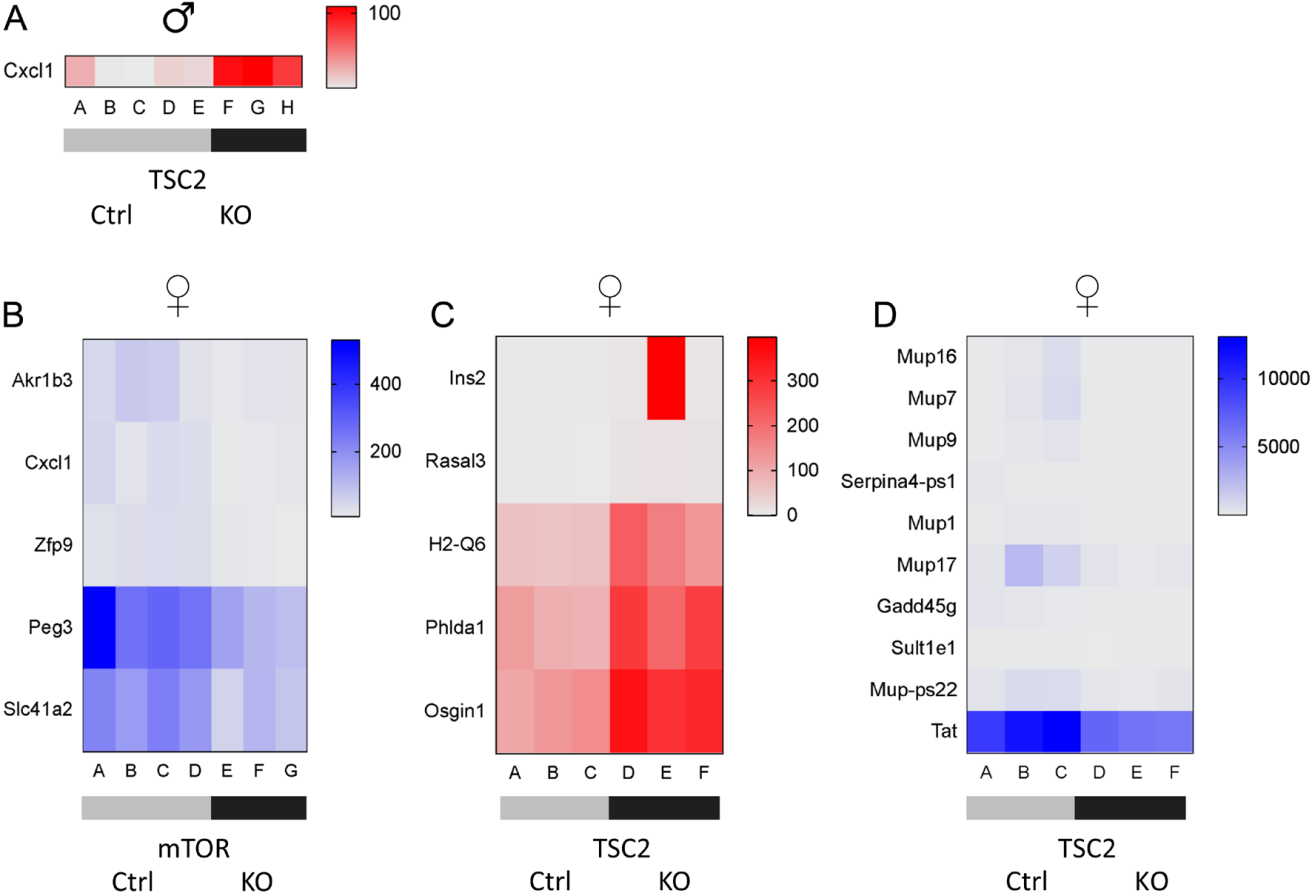
Liver RNA sequencing. (A) Heat map showing differentially expressed genes (DEGs) from male TSC2 control (*n* = 5) versus TSC2KO^pl^ (*n* = 3) liver comparison. Red indicates increased expression in the knockout offspring. (B) Heat map of genes with decreased expression (blue) from female mTOR control (*n* = 4) versus mTORKO^pl^ (*n* = 3) liver comparison. (C) Heat map of genes with increased expression (red) from female TSC2 control (*n* = 3) versus TSC2KO^pl^ (*n* = 3) liver comparison. (D) Heat map of genes with decreased expression (blue) from female TSC2 control (*n* = 3) versus TSC2KO^pl^ (*n* = 3) liver comparison. DEGs represent genes with fold-change greater than 2 and FDR < 0.05.

Insulin/Akt and mTOR/pS6 signaling were significantly altered in the livers of mTORKO^pl^ male and female offspring, respectively. Therefore, we performed RNA sequencing on the livers from mTORKO^pl^ and TSC2KO^pl^ adult offspring on an NCD. Our first analysis compared mTORKO^pl^ or TSC2KO^pl^ to their respective littermate controls. These pairwise comparisons yielded few DEGs. With a stringent two-fold change as the criteria and FDR of FDR < 0.05, we identified zero DEG in our mTORKO^pl^ male offspring liver comparison and one DEG in our TSC2KO^pl^ male offspring liver comparison (Fig. 3A). *Cxcl1*, a chemokine with a role in recruitment of neutrophils, was upregulated 5.69-fold in the TSC2KO^pl^ male offspring liver compared to littermate controls, which was confirmed by validation RT-qPCR (Supplementary Fig. 7A). We found that a total of five known genes were decreased in the mTORKO^pl^ female offspring liver compared to littermate controls (Supplementary Fig. 3B). *Slc41a2*, a plasma membrane magnesium transporter that was previously suggested to be a genetic variant associated with serum magnesium levels and risk for pre-diabetes (Kieboom *et al.* 2017), was validated by RT-qPCR (Supplementary Fig. 7B). A total of five known genes were upregulated (Fig. 3C) and ten known genes were downregulated (Fig. 3D) in the TSC2KO^pl^ female offspring liver compared to littermate controls. Fold-change values and FDR p-values can be found in Table 1.

**Table 1:** Fold-change and FDR *P* values of offspring liver DEGs.

Gene	EDGE test fold-change	FDR *P*-value
Cxcl1	5.698	0.00000001
Akr1b3	−3.294	0.0488
Cxcl1	−3.063	0.0077
Zfp9	−3.011	0.0058
Peg3	−2.964	0.0016
Slc41a2	−2.608	0.0002
Ins2	108.9	0.0495
Rasal3	6.301	0.0495
H2-Q6	2.552	0.0033
Phlda1	2.309	0.0093
Osgin1	2.282	0.0006
Mup16	−29.11	0.0001
Mup7	−23.06	0.0001
Mup9	−17.68	0.00000066
Serpina4-ps	−9.427	0.000035
Mup1	−7.756	0.0001
Mup17	−6.961	0.0264
Gadd45g	−4.989	0.0006
Sult1e1	−3.768	0.0207
Mup-ps22	−3.509	0.0294
Tat	−1.965	0.0092

We further analyzed our RNA sequencing data by comparing the livers of mTORKO^pl^ to TSC2KO^pl^ offspring. We found 168 DEGs between mTORKO^pl^ versus TSC2KO^pl^ male offspring liver. A total of 98 genes were upregulated in the TSC2KO^pl^ male liver compared to the mTORKO^pl^ male liver, whereas 70 genes were downregulated (Supplementary Fig. 8A and B). Furthermore, we found that 191 genes were differentially expressed in the mTORKO^pl^ versus TSC2KO^pl^ female offspring liver. Expression levels of 115 genes were increased in the TSC2KO^pl^ female liver compared to the mTORKO^pl^ female liver, whereas 76 genes were decreased (Supplementary Fig. 9A and B). Next, we performed Gene Ontology, KEGG analysis and Ingenuity Pathway Analysis on DEGs from the male and female liver (Supplementary Table 1). In the female liver, genes within processes involving glucose homeostasis, lipid metabolism, redox processes, and mitochondrion morphogenesis were differentially expressed. DEGs identified in the female liver were involved in pathways associated with steroid hormone biosynthesis, metabolism, and the phagosome. Glucose and lipid metabolism and regulation of hormone levels processes were also observed in the male liver, with the addition of neurotransmitter transport and cell–cell signaling. DEGs from the female liver were more broadly associated with endocrine system disorders and metabolic disease, whereas the top disease associated with DEGs from the male liver was cancer (Supplementary Table 1).

### Mup1 gene expression is differentially altered in female mTORKO^pl^ and TSC2KO^pl^ offspring livers

**Figure 4:**
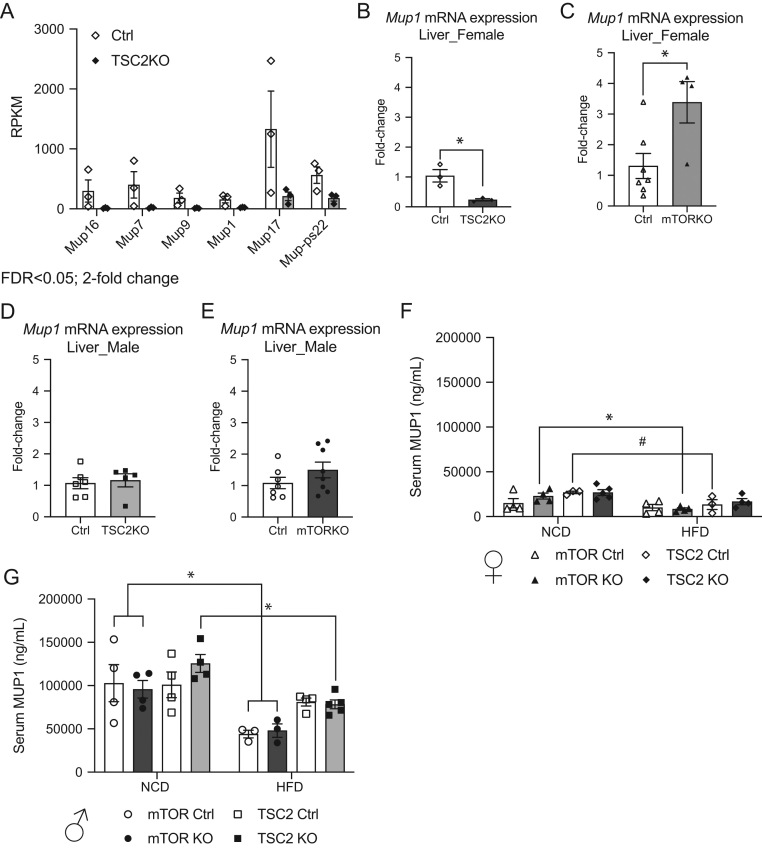
Mup1 levels in the liver and serum of placental mTORKO^pl^ and TSC2KO^pl^ adult offspring. (A) Comparison of reads for various Mup family genes detected in RNA sequencing analysis of TSC2KO^pl^ female liver versus littermate controls. All Mup genes had fold-change > 2 and FDR < 0.05. Hepatic *Mup1* mRNA expression of 90-day-old adult (B) TSC2KO^pl^ females compared to littermate controls (*n* = 3), (C) mTORKO^pl^ females compared to littermate controls (*n* = 4–7), (D) TSC2KO^pl^ males compared to littermate controls (*n* = 5–6), (E) mTORKO^pl^ males compared to littermate controls (*n* = 7–8). (F) Circulating Mup1 levels in placental mTORKO^pl^ (*n* = 4) and TSC2KO^pl^ (*n* = 3–5) female offspring on normal chow diet and high-fat diet and respective littermate controls. (G) Circulating Mup1 levels in placental mTORKO^pl^ (*n* = 4) and TSC2KO^pl^ (*n* = 3–4) male offspring on normal chow diet and high-fat diet and respective littermate controls. Statistical analyses were conducted using an unpaired two-tailed *t*-test and repeated measures two-way ANOVA, with significance *P < 0.05, ^#^*P* < 0.1.

Within our RNA sequencing comparisons, we found that several members of a family of genes called major urinary protein (*Mup*) genes were downregulated in the TSC2KO^pl^ female offspring liver (*Mup16, Mup7, Mup9, Mup1, Mup17;* Fig. 4A). *Mup*1 is the main and most well-studied member of the MUP family of proteins. MUPs are lipocalins that historically function as pheromone transporters and have been implicated to regulate glucose homeostasis (Zhou & Rui 2010).

Importantly, in agreement with our RNA sequencing data, we validated a significant decrease in *Mup1* mRNA level in the livers of TSC2KO^pl^ female offspring (Fig. 4B). Conversely, we found a significant increase in *Mup1* mRNA level in the livers of placental mTORKO female offspring (Fig. 4C). We did not detect differences in *Mup1* mRNA level in the livers of TSC2KO^pl^ or mTORKO^pl^ male offspring compared to their respective controls (Fig. 4D and E). These gene expression data suggest that *Mup1* differences are specific to the female offspring liver.

MUP1 is generated and secreted from the liver into the circulation. Therefore, we measured serum levels of MUP1 in mice on an NCD. Serum MUP1 levels were significantly lower in female offspring compared to males (Supplementary Fig. 10A), consistent with previous reports (Hui *et al.* 2009). Serum MUP1 was not different in the TSC2KO^pl^ or mTORKO^pl^ female offspring compared to controls on NCD (Fig. 4E, NCD). Similarly, serum MUP1 was not different among genotypes in male offspring on an NCD (Fig. 4F, NCD).

### Serum and hepatic MUP1 levels in response to high-fat diet, fasting, and insulin stimulation

Studies have shown that Mup1 levels are altered in response to nutritional status (Hui *et al.* 2009, Zhou & Rui 2010). Therefore, we tested if HFD feeding could impact serum levels of Mup1 in our models. Female TSC2KO^pl^ and mTORKO^pl^ offspring displayed no differences compared to respective controls in serum MUP1 when subjected to an HFD (Fig. 4E, HFD). Serum MUP1 was not different between controls and knockouts from male mTORKO^pl^ and TSC2KO^pl^ animals on HFD (Fig. 4F, HFD). In previous studies by other groups, MUP1 was shown to be decreased in the HFD condition (Hui *et al.* 2009, Zhou *et al.* 2009). In the current study, we found generally lower levels of MUP1 in the serum of males on HFD compared to NCD (Fig. 4F, NCD vs HFD), except for TSC2 control males with no difference in serum MUP1 between NCD and HFD. Only mTORKO^pl^ female offspring had significantly reduced serum MUP1 in HFD compared to NCD (Fig. 4E, NCD vs HFD, filled triangles).

Past studies indicate that fasting induces a reduction in *Mup1* mRNA expression in the liver (Hui *et al.* 2009, Zhou *et al.* 2009). Presumably, hepatic protein expression of MUP1 is also reduced in the fasted state, making MUP1 differences at the protein level potentially challenging to detect, especially in female offspring where MUP1 expression is lower compared to males. Nonetheless, we measured MUP in the livers of 10-h fasted animals. At the protein level, no differences in Mup1 were observed in mTORKO^pl^ female offspring liver compared to littermate controls (Supplementary Fig. 10B, quantified in Supplementary Fig. 10C). No differences in MUP protein in TSC2KO^pl^ female offspring liver compared to controls were detected (Supplementary Fig. 10B, quantified in Supplementary Fig. 10D). There were no differences in MUP in the livers of mTORKO^pl^ or TSC2KO^pl^ male offspring (Supplementary Fig. 10B, quantified in Supplementary Fig. 10E and F), consistent with mRNA levels in the fed state. For circulating levels of MUP1 during fasting, we found that mTORKO^pl^ female offspring do not display significant differences in MUP1 (Supplementary Fig. 10G), whereas TSC2KO^pl^ female offspring have significantly lowered MUP1 compared to respective littermate controls (Supplementary Fig. 10H). Serum MUP1 in fasted male offspring was not different by genotype (Supplementary Fig. 10I and J).

We next assessed whether local hepatic MUP protein was altered in response to insulin stimulation in the mTORKO^pl^ female offspring compared to the mTOR control female offspring. We focused on the female offspring liver because we saw female-specific differences in *Mup1* mRNA expression in non-insulin-treated liver samples. No differences in insulin-mediated MUP expression were detected (Supplementary Fig. 10K), indicating that acute insulin treatment as well as corresponding activation of hepatic insulin signaling does not regulate local MUP expression in the female offspring liver.

### Nutrient sensing-related regulation of Mup1 in murine hepatocyte cell line

**Figure 5:**
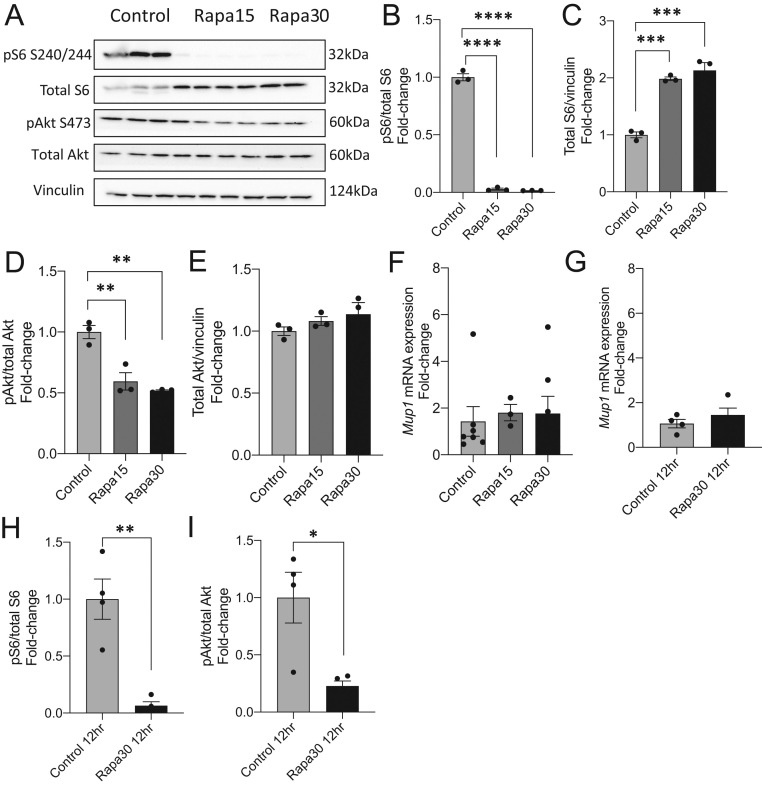
Mup1 mRNA is not altered by inhibited mTOR-S6 signaling in rapamycin-treated AML12 hepatocyte cells. (A) Western blots of phosphorylated S6 at serine 240/244, total S6, phosphorylated Akt at serine 473, total Akt, and vinculin from AML12 hepatocyte cells treated with 15 nM or 30 nM rapamycin for 24 h. (B) Quantification of pS6/total S6 represented as fold-change relative to control for 24-h rapamycin-treated cells (*n* = 3). (C) Quantification of pAkt/total Akt represented as fold-change relative to control for 24-h rapamycin-treated cells (*n* = 3). (D) Quantification of total S6/vinculin represented as fold-change relative to control for 24-h rapamycin-treated cells (*n* = 3). (E) Quantification of total Akt/vinculin represented as fold-change relative to control for 24-h rapamycin-treated cells (*n* = 3). (F) *Mup1* mRNA expression of AML12 hepatocyte cells treated for 24 h with 15 nM or 30 nM rapamycin. (G) *Mup1* mRNA expression of AML12 hepatocyte cells treated for 12 h with 30 nM rapamycin. Quantification of Western blots for (H) phosphorylated S6 and (I) phosphorylated Akt represented as fold-change relative to control (*n* = 3) for 30 nM rapamycin treatment for 12 h. Statistical analyses were conducted using an unpaired two-tailed *t*-test or a one-way ANOVA with Tukey’s *post hoc* test, with significance *P < 0.05, **P < 0.01, ***P < 0.001, and ****P < 0.0001.

Because *Mup1* mRNA expression and mTOR nutrient signaling were differentially altered in the livers of female offspring that experienced manipulation of placental mTOR during fetal development, we next evaluated whether there was a relationship between mTOR signaling and *Mup1* gene expression in the liver. Based on our findings that mTOR signaling and *Mup1* mRNA are increased in the mTORKO^pl^ female offspring liver compared to controls, we hypothesized that inhibiting mTOR signaling would decrease *Mup1* expression if mTOR directly regulates *Mup1* expression. First, we used the AML12 murine hepatocyte cell line to test whether inhibited mTOR nutrient sensing was associated with changes in *Mup1* expression. AML12 cells were treated with rapamycin (15 nM and 30 nM concentrations) for 24 h to pharmacologically inhibit mTORC1. These concentrations and timing were used to inhibit mTORC1 without impacting mTORC2. We confirmed robust inhibition of mTOR signaling by measuring phosphorylation of ribosomal protein S6 in rapamycin-treated cells (Fig. 5A, quantified in Fig. 5B). Rapamycin treatment increased total S6 protein levels (Fig. 5A, quantified in Fig. 5C). We observed that rapamycin treatment resulted in significantly decreased phosphorylated Akt to 50% of controls (Fig. 5A, quantified in Fig. 5D) without changes to total Akt protein (Fig. 5A, quantified in Fig. 5E). Upon confirming inhibition of mTOR signaling in the rapamycin-treated cells, we next measured mRNA level of *Mup1*. We determined that 15 nM and 30 nM rapamycin treatment for 24 h conferred no changes in *Mup1* expression (Fig. 5F). We postulated that the 24-h time point may be too long to observe our hypothesized reduction in *Mup1*. Therefore, we treated AML12 cells with 30 nM rapamycin for 12 h. However, we saw no differences in *Mup1* expression at the 12-h timepoint (Fig. 5G), despite depleted mTOR/S6 signaling and significantly reduced Akt signaling (Fig. 5H and I).

### Acute rapamycin treatment *in vivo* does not alter serum MUP1 in adult female mice

**Figure 6:**
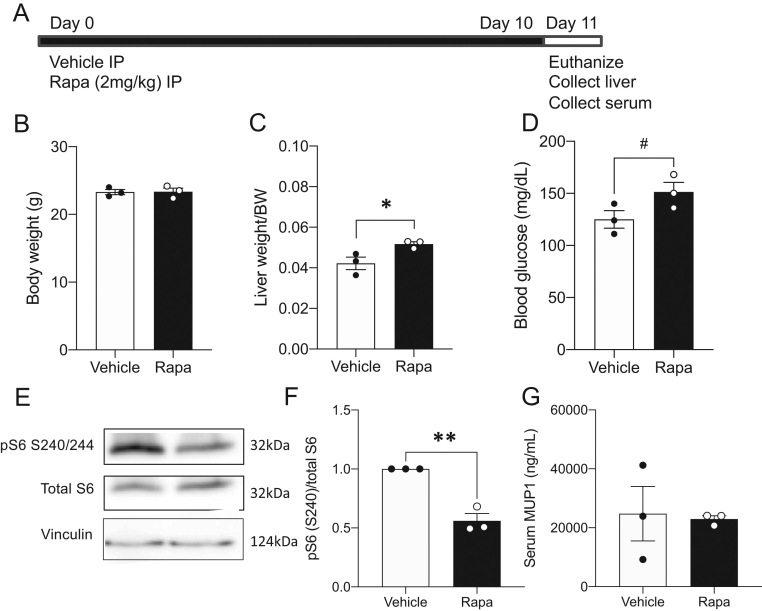
Acute rapamycin treatment* in vivo* reduces hepatic mTOR signaling with no impact on circulating Mup1. (A) Timeline of 2 mg/kg body weight intraperitoneal administration of rapamycin or saline as vehicle followed by the collection of tissue and serum. Posttreatment (B) body weight, (C) liver weight relative to body weight, and (D) blood glucose measurements. (E) Representative Western blot of phosphorylated S6 at serine 240/244, total S6, and vinculin from livers of vehicle- or rapamycin-treated females. (F) Quantification of pS6/total S6 represented as fold-change relative to vehicle (*n* = 3). (G) Circulating MUP1 levels in females at the end of 10-day treatment with the vehicle of rapamycin (*n* = 3). Statistical analyses were conducted using an unpaired two-tailed *t*-test, with significance *P < 0.05, **P < 0.01, and ^#^ *P* < 0.1.

To characterize the effect of acute rapamycin treatment *in vivo*, we performed daily intraperitoneal injections of 2 mg/kg rapamycin for 10 days and subsequently collected liver tissue and serum on day 11 (Fig. 6A). Following the acute rapamycin treatment, body weight was not altered but liver weight relative to body weight was increased (Fig. 6B and C). Blood glucose levels trended higher in females treated with rapamycin (Fig. 6D). As expected with rapamycin treatment, mTORC1 target phospho-S6 was significantly reduced in the liver (Fig. 6E, quantified in Fig. 6F) without impacting circulating MUP1 levels in the serum (Fig. 6G).

## Discussion

We sought to test how manipulation of placental mTOR signaling *in utero* alters fetal programming of the gene expression and nutrient sensing responses of peripheral tissues important for maintaining glucose homeostasis. We found that the liver of mTORKO^pl^ offspring displayed altered programming of nutrient signaling pathways (Akt and S6) in a sexually dimorphic manner. In response to insulin stimulation, the male mTORKO^pl^ offspring liver shows elevated phosphorylated Akt. The female offspring mTORKO^pl^ liver has enhanced phosphorylated S6 both in nonstimulated and insulin-stimulated conditions. RNA sequencing comparisons suggest a malprogramming in liver tissue occurred prior to a metabolic challenge. We identified *Mup1* as a gene candidate potentially regulated by nutrient changes, namely fasting and HFD.

Body weight and blood glucose levels in this study recapitulated our previously published data where mTORKO^pl^ and TSC2KO^pl^ male and female offspring display no differences in body weight at 12 weeks on an NCD (Akhaphong *et al.* 2021). Our previous study thoroughly characterized glucose and insulin tolerance, glucose-stimulated insulin secretion (*in vitro* and *in vivo*), and beta-cell mass in placental mTOR and TSC2 models on NCD and HFD. *In vivo* phenotyping revealed normal glucose tolerance for male and female mTORKO^pl^ or TSC2KO^pl^ offspring in NCD. Insulin sensitivity was normal in male mTORKO^pl^ offspring on an NCD and improved in male TSC2KO^pl^ offspring. Insulin sensitivity was normal in both female mTORKO^pl^ or TSC2KO^pl^ offspring. Subtle phenotypic differences when exposed to an NCD precede robust metabolic alterations upon challenge with an HFD. Therefore, we sought to determine how perturbations in placental nutrient signaling impact baseline programming of offspring nutrient-sensing pathways without HFD challenge. Glucose homeostasis is not only controlled by the insulin-secreting beta-cells but also by peripheral tissues important for the storage, production, and metabolism of glucose. Therefore, we turned to the liver, adipose tissue, and skeletal muscle to assess insulin signaling and nutrient-sensing pathways.

The relationship between maternal–fetal nutrition and insulin signaling in offspring peripheral tissues has been previously investigated. Manipulation of the maternal diet, such as introducing a low-protein diet or HFD during gestation, can impact the offspring peripheral tissues. For example, maternal obesity results in male offspring hyperinsulinemia and insulin resistance by 3 months of age, accompanied by increased phospho-IRS1 S307 (decreased insulin signaling activity) and reduced phospho-Akt S473 in the offspring liver (Martin-Gronert *et al.* 2010). Maternal overnutrition decreased phospho-Akt S473 and increased total Akt in the soleus skeletal muscle of adult male rat offspring (Latouche *et al.* 2014). On the other hand, adult offspring from maternal low-protein diet had decreased IRS1 and phospho-AKT S473 in white adipose tissue (Martin-Gronert *et al.* 2016). The global physiological effect of this differential signaling has been shown to involve altered insulin sensitivity such that maternal obesity leads to insulin resistance and maternal low protein leads to increased insulin sensitivity (Alejandro *et al.* 2014). Because mTOR signaling is a nutrient sensor important for sufficient placental and fetal growth (Akhaphong *et al.* 2021), we expected our mTORKO^pl^ model to mimic low-protein diet outcomes.

In the present study, we found that the male mTORKO^pl^ offspring liver had elevated phosphorylated Akt, indicative of increased insulin signaling, in response to insulin stimulation compared to the male mTOR control liver. We interpreted these data in two possible ways: (i) Enhanced hepatic insulin signaling preceding a metabolic challenge may protect the male mTORKO^pl^ offspring from insulin resistance when subjected to the metabolic challenge of an HFD, as seen in Akhaphong *et al.* (Akhaphong *et al.* 2021); (ii) The increase in hepatic insulin signaling may be reflective of underlying dysfunction. However, insulin tolerance tests performed in male mTOR animals on an NCD crudely suggest no differences at the phenotypic level. We observed that the female mTORKO^pl^ offspring liver did not display altered Akt signaling but rather had enhanced phosphorylated S6, a downstream target of mTOR signaling, at baseline. Without similar changes in phospho-Akt, which is interrelated with mTOR signaling, our findings suggest activation of mTOR signaling independent of Akt. Furthermore, the enhanced basal level of phospho-S6 in the mTORKO^pl^ female offspring liver suggests a hyperactive nutrient sensing that seems to be unresponsive to insulin stimulation. Hyperactivation of S6 (and Akt) preceding insulin resistance has been observed in previous studies (Tremblay *et al.* 2005, Um *et al.* 2006, Mao & Zhang 2018). Interestingly, we observed molecular changes in tissues but did not detect differences in insulin tolerance at the whole animal level in offspring on an NCD. However, under hyper-nutrient environment such as HFD over a period of time, these animals display increased susceptibility to glucose intolerance associated with insulin resistance based on insulin tolerance test (Akhaphong *et al.* 2021). In our models, the male offspring may be primed differently to respond to insulin compared to female offspring on NCD, which may have made them more tolerant to the metabolic challenge of an HFD.

The role of MUP1 in metabolism is largely unexplored. Few studies implicate MUP1 to positively regulate glucose homeostasis as increased *Mup1* expression improves glucose tolerance in male *db/db* models (Hui *et al.* 2009, Zhou *et al.* 2009). It is thought to suppress glucose production by reducing gluconeogenic genes and inhibiting the hepatic lipogenic program (Hui *et al.* 2009, Zhou *et al.* 2009). MUP1 expression and circulating levels appear to be decreased in obesity and fasting states (Hui *et al.* 2009, Zhou *et al.* 2009) and reduced in females compared to males. Moreover, MUP1 has been reported to improve insulin sensitivity in the skeletal muscle at least partly through increasing insulin-stimulated Akt signaling and enhancing mitochondrial biogenesis (Hui *et al.* 2009). However, phosphorylation of the insulin receptor and Akt in the liver were similarly stimulated by insulin in MUP1 overexpression and controls (Zhou *et al.* 2009) suggesting that MUP1 does not alter hepatic insulin signaling in male *db/db* mice. In our study, we observed the expected reduction of serum MUP1 in response to an HFD in male offspring across genotypes. In contrast, this reduction was only evident in the mTORKO^pl^ female offspring who are metabolically worse than mTOR control female offspring and TSC2 female offspring on an HFD. While the reduction in serum MUP1 has been reported in male and female *db/db* mice (Hui *et al.* 2009), our data suggest that circulating MUP1 levels decline in females upon HFD-induced obesity. Furthermore, our data suggest that circulating MUP1 level in the female mTORKO^pl^ offspring appears to be responsive to nutrient status while the female controls and TSC2KO^pl^ female offspring are not. In fact, the decrease in circulating MUP1 levels in mTORKO^pl^ female offspring on an HFD mimics the male offspring; while we did not see differences in glucose or insulin tolerance by genotype in the male models, we know that males are typically more glucose and insulin intolerant than females in general (Mauvais-Jarvis 2018, Jo *et al.* 2023). Future studies are needed to understand why overexpression of MUP1 improves male metabolic phenotypes to such an extent when males have higher levels of MUP1 at baseline, but females, who have low levels of MUP1, are historically better off metabolically.

In our initial attempt to understand the mechanisms of reduced hepatic *Mup1* expression and corresponding circulating MUP1 levels, we sought to ascertain whether mTOR signaling in the liver could play a role. A study by Schloesser *et al.* found a positive correlation between hepatic MUP1 expression and mTOR signaling during an HFD feeding scheme (Schloesser *et al.* 2015). This is in line with our findings that hepatic *Mup1* expression and mTOR/S6 signaling change in the same direction in mTORKO^pl^ female offspring. However, using the mouse AML12 hepatocyte cell line, we blocked mTOR signaling with rapamycin and observed no change in *Mup1* mRNA levels. To supplement this cell line investigation, we tested rapamycin *in vivo* by administering acute rapamycin treatment to female mice to reduce hepatic mTOR signaling. Differences in circulating MUP1 were not detected in response to decreased hepatic mTOR. Together, our experiments using rapamycin to decrease hepatic mTOR *in vitro* and *in vivo* indicate that MUP1 is not altered by reducing hepatic mTOR signaling. The impact of increasing hepatic mTOR signaling on local and circulating MUP1 levels has not been explored in our study or others. Alternatively, it is conceivable that while mTOR signaling does not regulate MUP1 levels, MUP1 may impact hepatic mTOR signaling and subsequent metabolic outcomes.

Limitations of our study include variability in specific offspring tissues and feasibility to investigate overexpression of hepatic mTOR. Future studies are needed to understand the effects of hepatic *Mup1* during obesity *in vivo* and in obesogenic conditions *in vitro*. Mechanistic evidence of *Mup1* regulation, secretion from the liver, and effects on target tissues are warranted. While *Mup1* was pursued in the present study due to its relevance to metabolism, a limited number of other DEGs were detected in our RNA sequencing analyses. Future studies may assess possible mechanistic roles for other candidate DEGs. Considering the limited number of DEGs, regulatory mechanisms such as posttranscriptional modification could be explored, which may contribute to programming of insulin and nutrient signaling in offspring liver without robust changes in mRNA expression.

**Figure 7:**
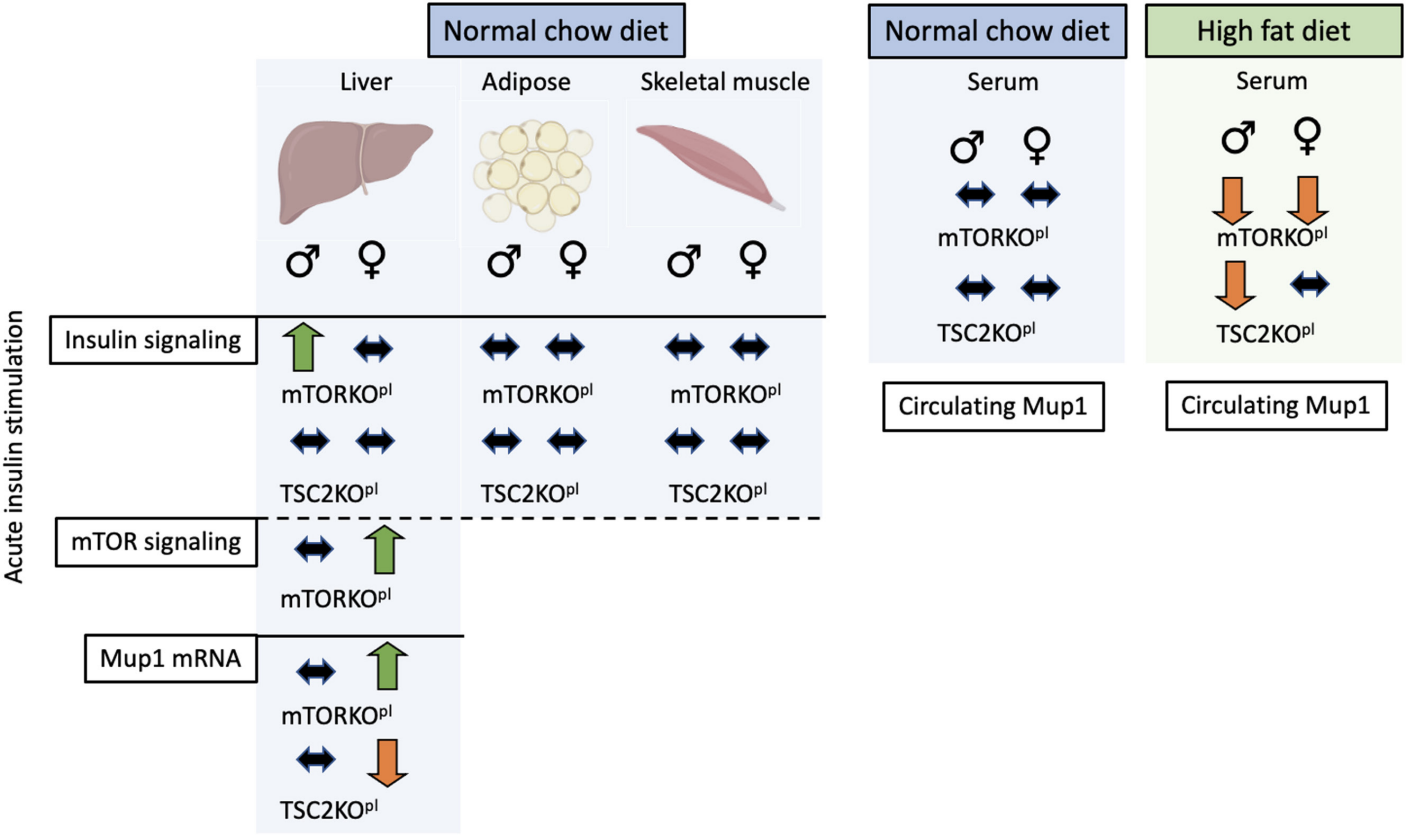
Overview of findings.

To summarize our study, we showed that programming of insulin signaling and nutrient-sensing pathways in the offspring liver is modulated by placental mTOR in a sexually dimorphic manner (Fig. 7A). Specifically in the female offspring, hyperactive hepatic nutrient sensing via mTOR signaling, local liver *Mup1* expression, and levels of circulating MUP1 may underlie the programmed response to nutritional status and metabolic stress. Collectively, these data bolster our understanding of fetal programming of metabolic peripheral tissues which may contribute to the development of obesity, insulin resistance, and T2D.

## Supplementary Materials

Supplementary Material

## Declaration of interest

The authors declare no conflict of interest.

## Funding

The NIH funding for EUA (R01DK136237 and Regenerative Medicine Minnesota http://dx.doi.org/10.13039/100016944), University of Minnesota http://dx.doi.org/10.13039/100007249 Foundation, McKnight Foundation http://dx.doi.org/10.13039/100005270, and the UMN Genomics Center. Dr Megan Beetch was supported by T32DK007203 and T32DK083250, Ms Alicia Wong was supported by T32GM140936, and Mr Seokwon Jo by NIDDK F31DK131860. Ms Briana Clifton was supported by the University of Minnesota http://dx.doi.org/10.13039/100007249 Inclusive Excellence Cardiovascular Research Opportunity Program for Undergraduates funded by the American Heart Association http://dx.doi.org/10.13039/100000968 (#846148).

## Data availability statement

Data available at request.

## Ethical approval

The Institutional Animal Care and Use Committee at the University of Minnesota approved of all animal studies.

## Author contribution statement

Conceptualization: MB and EUA; methodology: MB, BA, BC, AW, SJ, and EUA; formal analysis and investigation: MB; RNA sequencing data preparation and analysis: MB, BA, RM, and JEA-L; data curation and visualization: MB; writing of original draft: MB and EUA; review and editing: MB, SJ, AW, BC, BA, and EUA; supervision: EUA; funding acquisition: MB and EUA. All authors have read and agreed to the published version of the manuscript.
