# Supplementary material for: Impact of placental mTOR deficiency on peripheral insulin signaling in adult mice offspring
Source: J Mol Endocrinol. 2023 Oct 18;71(4):e230035. doi: 10.1530/JME-23-0035 (PMC10620464; doi:10.1530/JME-23-0035)
Supplement: Supplementary Material [file supplementary_material.pdf]

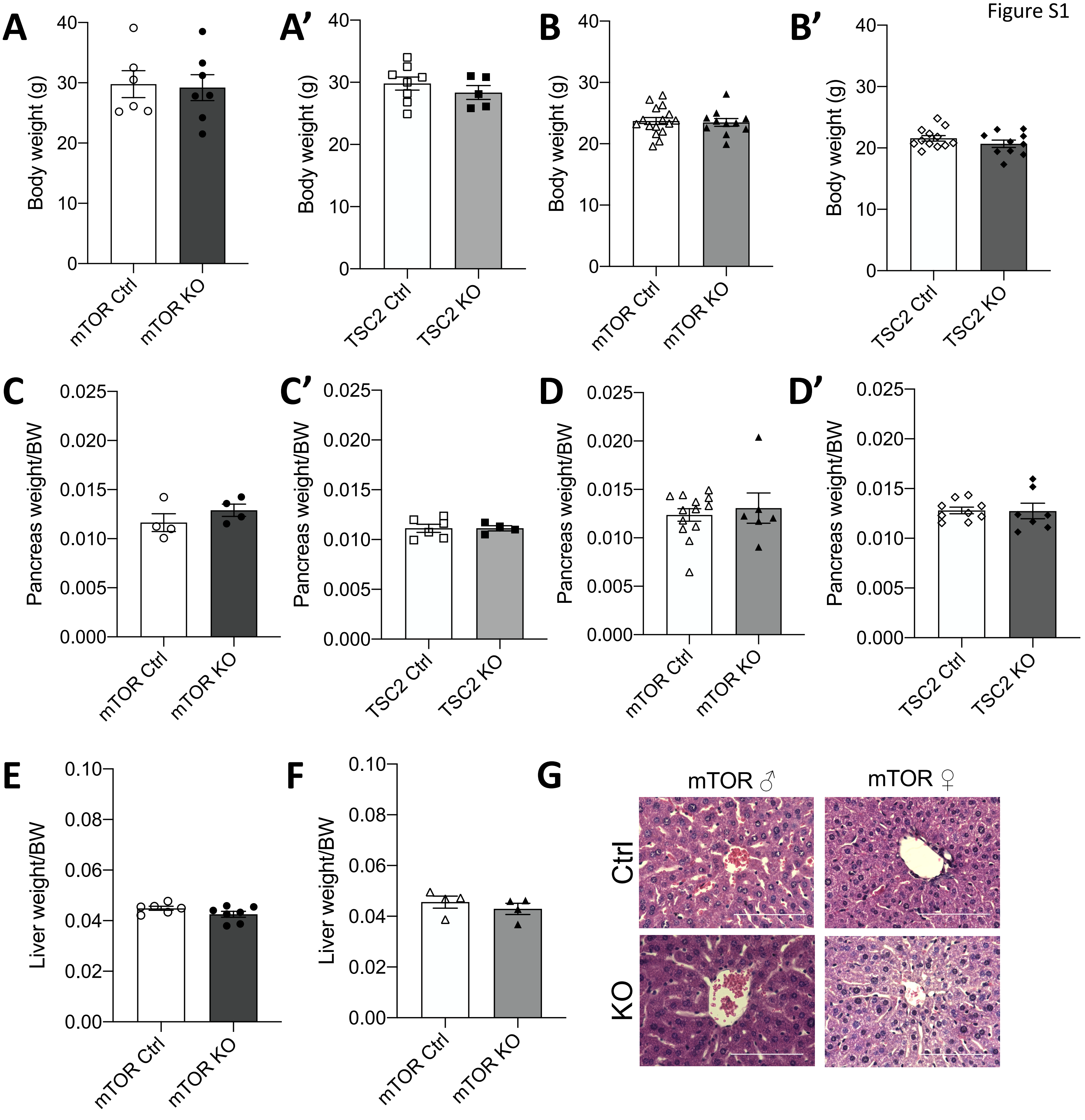

S. Fig. 1. Body weight and pancreas weight of placental mTORKO^{pl} and TSC2KO^{pl} offspring and littermate controls. Body weights of adult (**A**) male mTORKO^{pl} and littermate controls (n=6-7), (**A'**) male TSC2KO^{pl} and littermate controls (n=5-8), (**B**) female mTORKO^{pl} and littermate controls (n=11-16), and (**B'**) female TSC2KO^{pl} and littermate controls (n=10-12). Pancreas weight normalized by body weight of adult (**C**) male mTORKO^{pl} and littermate controls (n=4), (**C'**) male TSC2KO^{pl} and littermate controls (n=4-6), (**D**) female mTORKO^{pl} and littermate controls (n=9-14), and (**D'**) female TSC2KO^{pl} and littermate controls (n=8-11). Liver weight normalized by body weight of adult (**E**) male mTORKO^{pl} and littermate controls (n=6-7) and (**F**) female mTORKO^{pl} and littermate controls (n=4). (**G**) Images of H&E staining performed on liver tissues from mTORKO^{pl} male and female offspring. Statistical analyses were conducted using an unpaired two-tailed t-test, with significance *p<0.05.

Figure S2

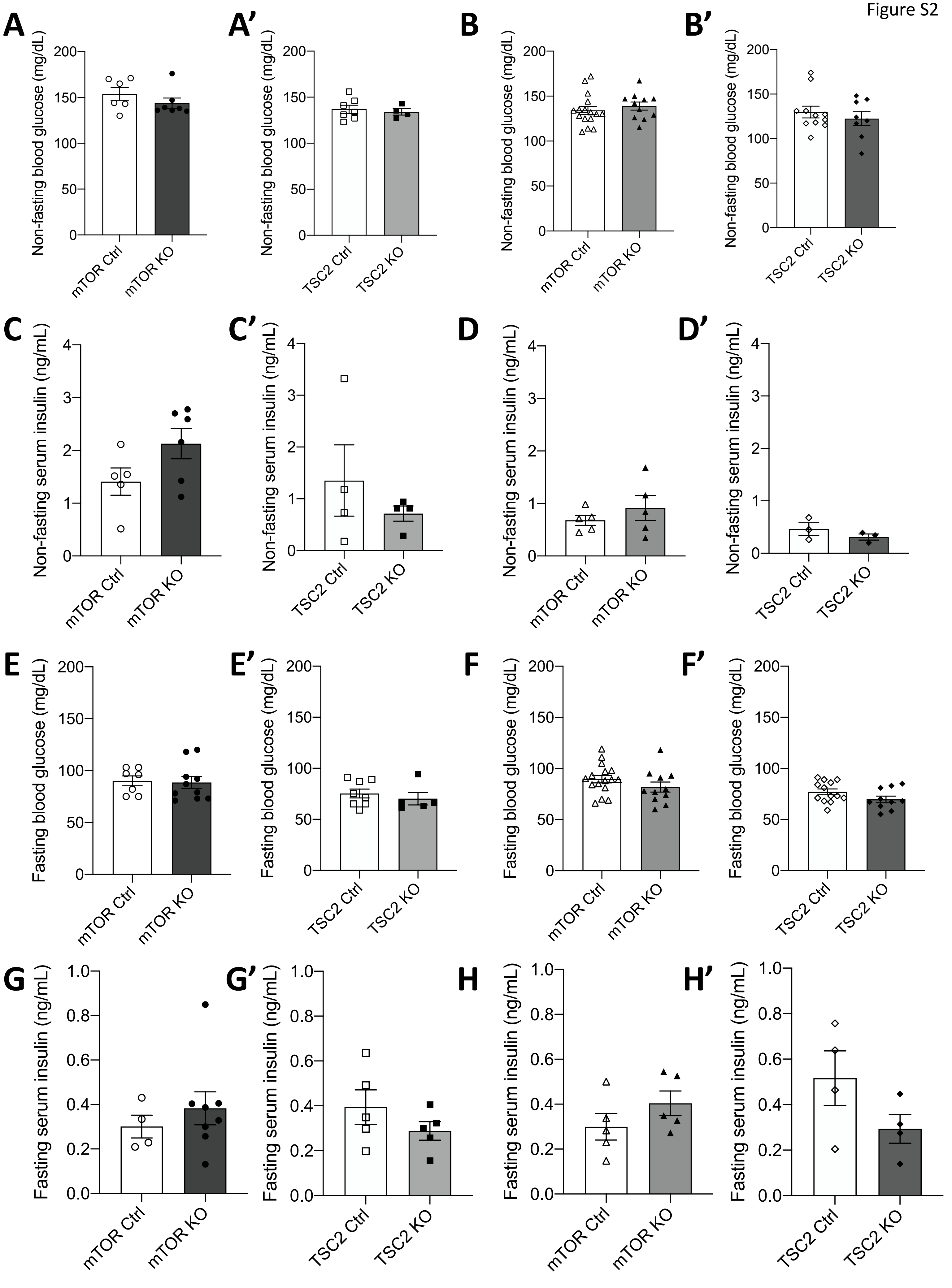
A
Non-fasting blood glucose (mg/dL)
0
50
100
150
200
mTOR Ctrl
mTOR KO
A'
Non-fasting blood glucose (mg/dL)
TSC2 Ctrl
TSC2 KO
B
Non-fasting blood glucose (mg/dL)
mTOR Ctrl
mTOR KO
B'
Non-fasting blood glucose (mg/dL)
TSC2 Ctrl
TSC2 KO
C
Non-fasting serum insulin (ng/mL)
0
1
2
3
4
mTOR Ctrl
mTOR KO
C'
Non-fasting serum insulin (ng/mL)
TSC2 Ctrl
TSC2 KO
D
Non-fasting serum insulin (ng/mL)
mTOR Ctrl
mTOR KO
D'
Non-fasting serum insulin (ng/mL)
TSC2 Ctrl
TSC2 KO
E
Fasting blood glucose (mg/dL)
0
50
100
150
200
mTOR Ctrl
mTOR KO
E'
Fasting blood glucose (mg/dL)
TSC2 Ctrl
TSC2 KO
F
Fasting blood glucose (mg/dL)
mTOR Ctrl
mTOR KO
F'
Fasting blood glucose (mg/dL)
TSC2 Ctrl
TSC2 KO
G
Fasting serum insulin (ng/mL)
0.0
0.2
0.4
0.6
0.8
1.0
mTOR Ctrl
mTOR KO
G'
Fasting serum insulin (ng/mL)
TSC2 Ctrl
TSC2 KO
H
Fasting serum insulin (ng/mL)
mTOR Ctrl
mTOR KO
H'
Fasting serum insulin (ng/mL)
TSC2 Ctrl
TSC2 KO

S. Fig. 2. Blood glucose and serum insulin levels of placental mTORKO^{pl} and TSC2KO^{pl} offspring and littermate controls. Non-fasting blood glucose of 90-day old (**A**) male mTORKO^{pl} and littermate controls (n=6-7), (**A'**) male TSC2KO^{pl} and littermate controls (n=4-6), (**B**) female mTORKO^{pl} and littermate controls (n=11-16), and (**B'**) female TSC2KO^{pl} and littermate controls (n=8-11). Non-fasting serum insulin of 90-day old (**C**) male mTORKO^{pl} and littermate controls (n=5-6), (**C'**) male TSC2KO^{pl} and littermate controls (n=4), (**D**) female mTORKO^{pl} and littermate controls (n=5), and (**D'**) female TSC2KO^{pl} and littermate controls (n=3). Fasting blood glucose of 90-day old (**E**) male mTORKO^{pl} and littermate controls (n=7-10), (**E'**) male TSC2KO^{pl} and littermate controls (n=5-8), (**F**) female mTORKO^{pl} and littermate controls (n=11-16), and (**F'**) female TSC2KO^{pl} and littermate controls (n=10-13). Fasting serum insulin of 90-day old (**G**) male mTORKO^{pl} and littermate controls (n=4-8), (**G'**) male TSC2KO^{pl} and littermate controls (n=5), (**H**) female mTORKO^{pl} and littermate controls (n=5), and (**H'**) female TSC2KO^{pl} and littermate controls (n=4). Statistical analyses were conducted using an unpaired two-tailed t-test with significance *$p<0.05$.

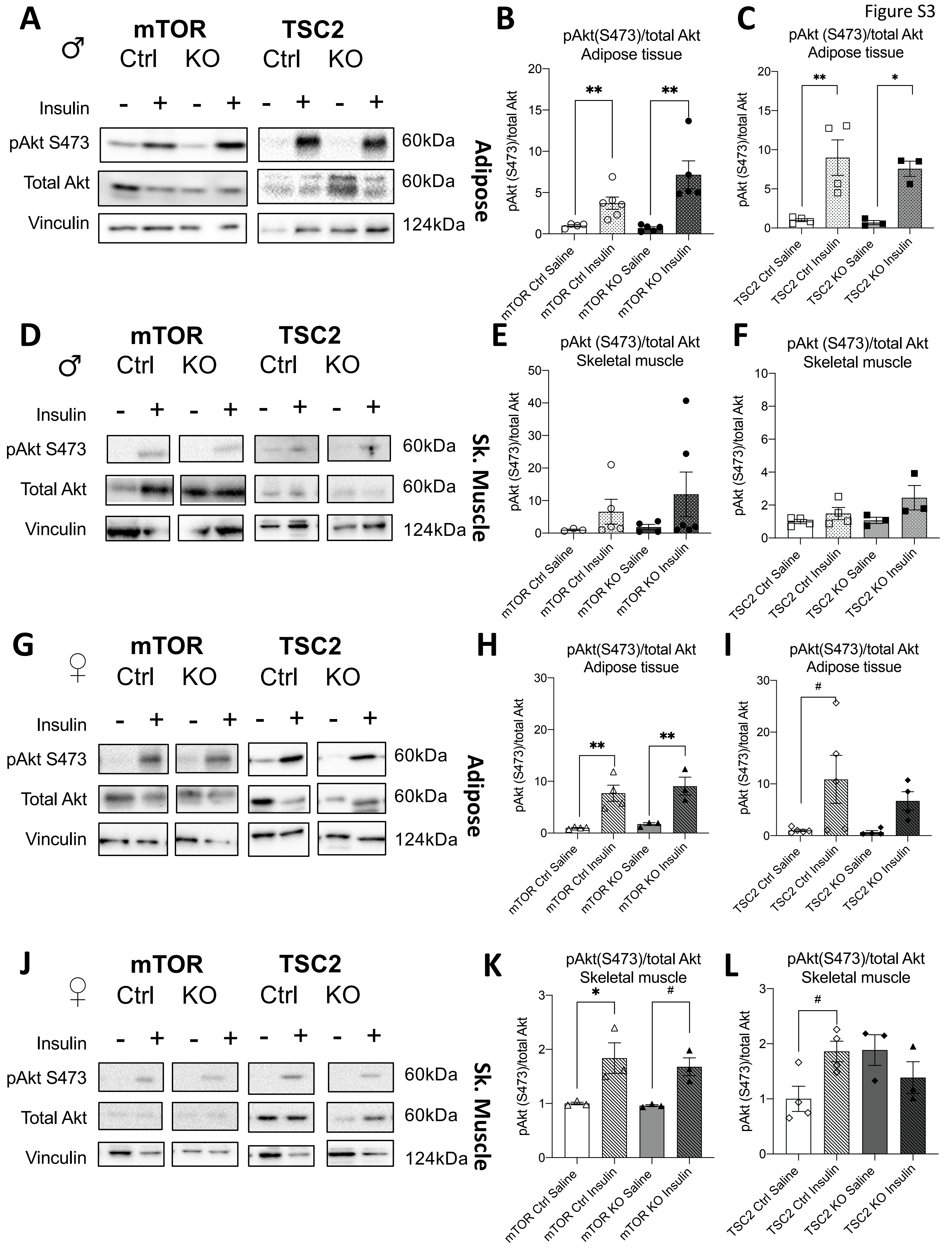
Figure S3
A
♂
mTOR
Ctrl KO
TSC2
Ctrl KO
Insulin
pAkt S473
Total Akt
Vinculin
60kDa
60kDa
124kDa
Adipose
B
pAkt(S473)/total Akt
Adipose tissue
C
pAkt (S473)/total Akt
Adipose tissue
pAkt (S473)/total Akt
mTOR Ctrl Saline
mTOR Ctrl Insulin
mTOR KO Saline
mTOR KO Insulin
TSC2 Ctrl Saline
TSC2 Ctrl Insulin
TSC2 KO Saline
TSC2 KO Insulin
D
Sk. Muscle
E
pAkt (S473)/total Akt
Skeletal muscle
F
pAkt (S473)/total Akt
Skeletal muscle
G
♀
H
pAkt(S473)/total Akt
Adipose tissue
I
pAkt(S473)/total Akt
Adipose tissue
J
K
pAkt(S473)/total Akt
Skeletal muscle
L
pAkt(S473)/total Akt
Skeletal muscle

S. Fig. 3. Insulin-Akt signaling in adipose tissue and skeletal muscle of placental mTORKO^{pl} and TSC2KO^{pl} adult offspring. (**A**) Representative Western blots of phosphorylated Akt at serine 473, total Akt, and vinculin from inguinal adipose tissue of 90-day-old mTOR control, mTORKO^{pl}, TSC2 control, and TSC2KO^{pl} male offspring injected with saline or 1 U/kg insulin. (**B**) Quantification of adipose tissue Western blots represented as fold-change relative to saline-treated mTOR control (n=4-6). (**C**) Quantification of adipose tissue Western blots represented as fold-change relative to saline-treated TSC2 control (n=3-4). (**D**) Representative Western blots of phosphorylated Akt at serine 473, total Akt, and vinculin from skeletal muscle of 90-day-old mTOR control, mTORKO^{pl}, TSC2 control, and TSC2KO^{pl} male offspring injected with saline or 1 U/kg insulin. (**E**) Quantification of skeletal muscle Western blots represented as fold-change relative to saline-treated mTOR control (n=3-6). (**F**) Quantification of skeletal muscle Western blots represented as fold-change relative to saline-treated TSC2 control (n=3-4). (**G**) Representative Western blots of phosphorylated Akt at serine 473, total Akt, and vinculin from inguinal adipose tissue of 90-day-old mTOR control, mTORKO^{pl}, TSC2 control, and TSC2KO^{pl} female offspring injected with saline or 1 U/kg insulin. (**H**) Quantification of adipose tissue Western blots represented as fold-change relative to saline-treated mTOR control (n=3-4). (**I**) Quantification of adipose tissue Western blots represented as fold-change relative to saline-treated TSC2 control (n=3-4). (**J**) Representative Western blots of phosphorylated Akt at serine 473, total Akt, and vinculin from skeletal muscle of 90-day-old mTOR control, mTORKO^{pl}, TSC2 control, and TSC2KO^{pl} female offspring injected with saline or 1 U/kg insulin. (**K**) Quantification of skeletal muscle Western blots represented as fold-change relative to saline-treated mTOR control (n=3). (**L**) Quantification of skeletal muscle Western blots represented as fold-change relative to saline-treated TSC2 control (n=3-4). Statistical analyses were conducted using one-way ANOVA with Tukey's post-hoc test, with significance *$p<0.05$.

Figure S4

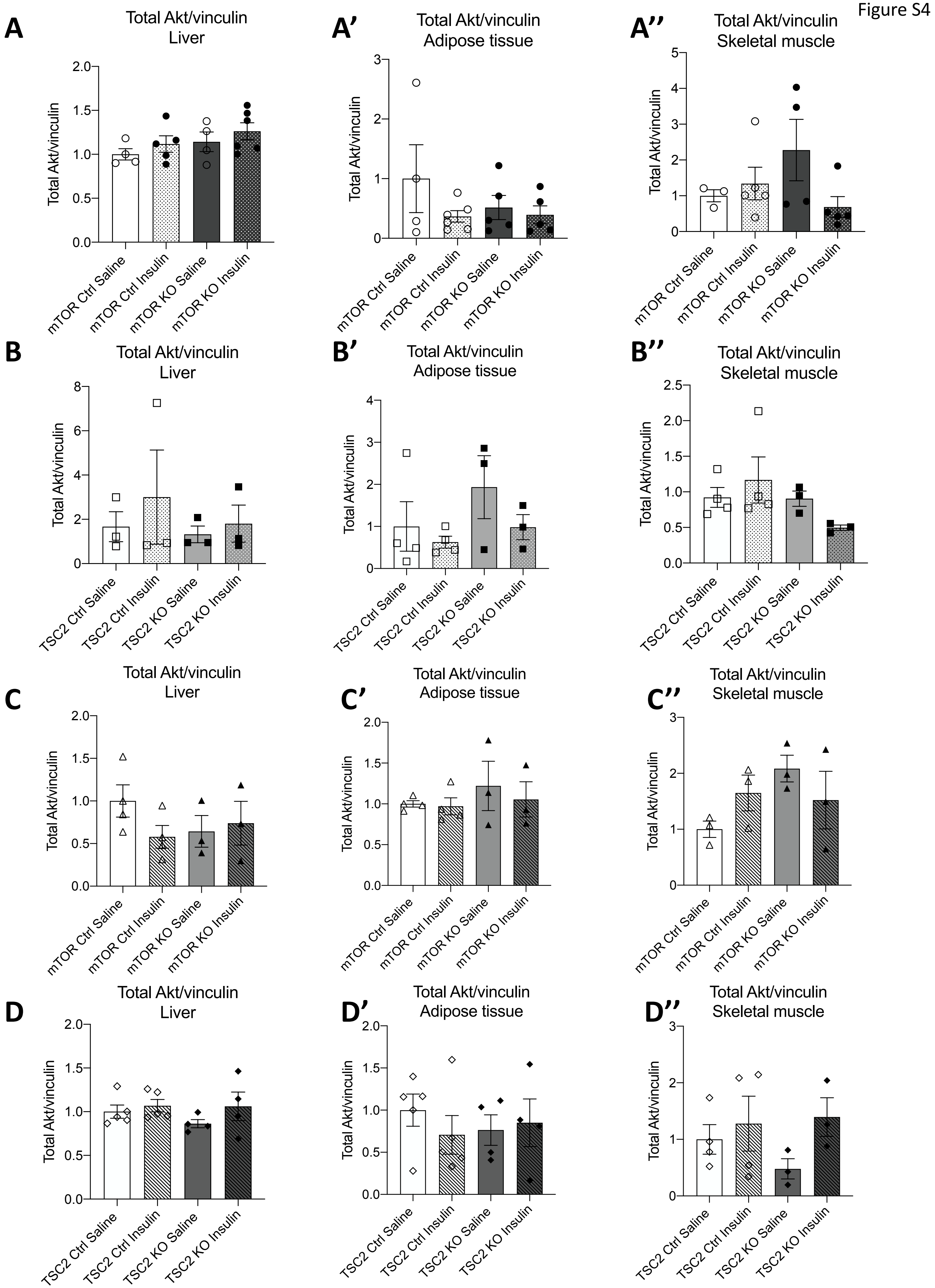

A
Total Akt/vinculin
Liver
A'
Total Akt/vinculin
Adipose tissue
A''
Total Akt/vinculin
Skeletal muscle
B
Total Akt/vinculin
Liver
B'
Total Akt/vinculin
Adipose tissue
B''
Total Akt/vinculin
Skeletal muscle
C
Total Akt/vinculin
Liver
C'
Total Akt/vinculin
Adipose tissue
C''
Total Akt/vinculin
Skeletal muscle
D
Total Akt/vinculin
Liver
D'
Total Akt/vinculin
Adipose tissue
D''
Total Akt/vinculin
Skeletal muscle
Total Akt/vinculin
mTOR Ctrl Saline
mTOR Ctrl Insulin
mTOR KO Saline
mTOR KO Insulin
TSC2 Ctrl Saline
TSC2 Ctrl Insulin
TSC2 KO Saline
TSC2 KO Insulin

S. Fig. 4. Total Akt levels in liver, adipose, and skeletal muscle of placental mTORKO^{pl} and TSC2KO^{pl} offspring and littermate controls. Quantification of total Akt normalized to vinculin loading control in (**A**) liver, (**A'**) adipose tissue, and (**A"**) skeletal muscle of male mTORKO^{pl} and littermate controls with and without insulin stimulation (n=3-6). Quantification of total Akt normalized to vinculin loading control in (**B**) liver, (**B'**) adipose tissue, and (**B"**) skeletal muscle of male TSC2KO^{pl} and littermate controls with and without insulin stimulation (n=3-4). Quantification of total Akt normalized to vinculin loading control in (**C**) liver, (**C'**) adipose tissue, and (**C"**) skeletal muscle of female mTORKO^{pl} and littermate controls with and without insulin stimulation (n=3-4). Quantification of total Akt normalized to vinculin loading control in (**D**) liver, (**D'**) adipose tissue, and (**D"**) skeletal muscle of female TSC2KO^{pl} and littermate controls with and without insulin stimulation (n=3-5). Statistical analyses were conducted using one-way ANOVA with Tukey's post-hoc test, with significance *$p<0.05$.

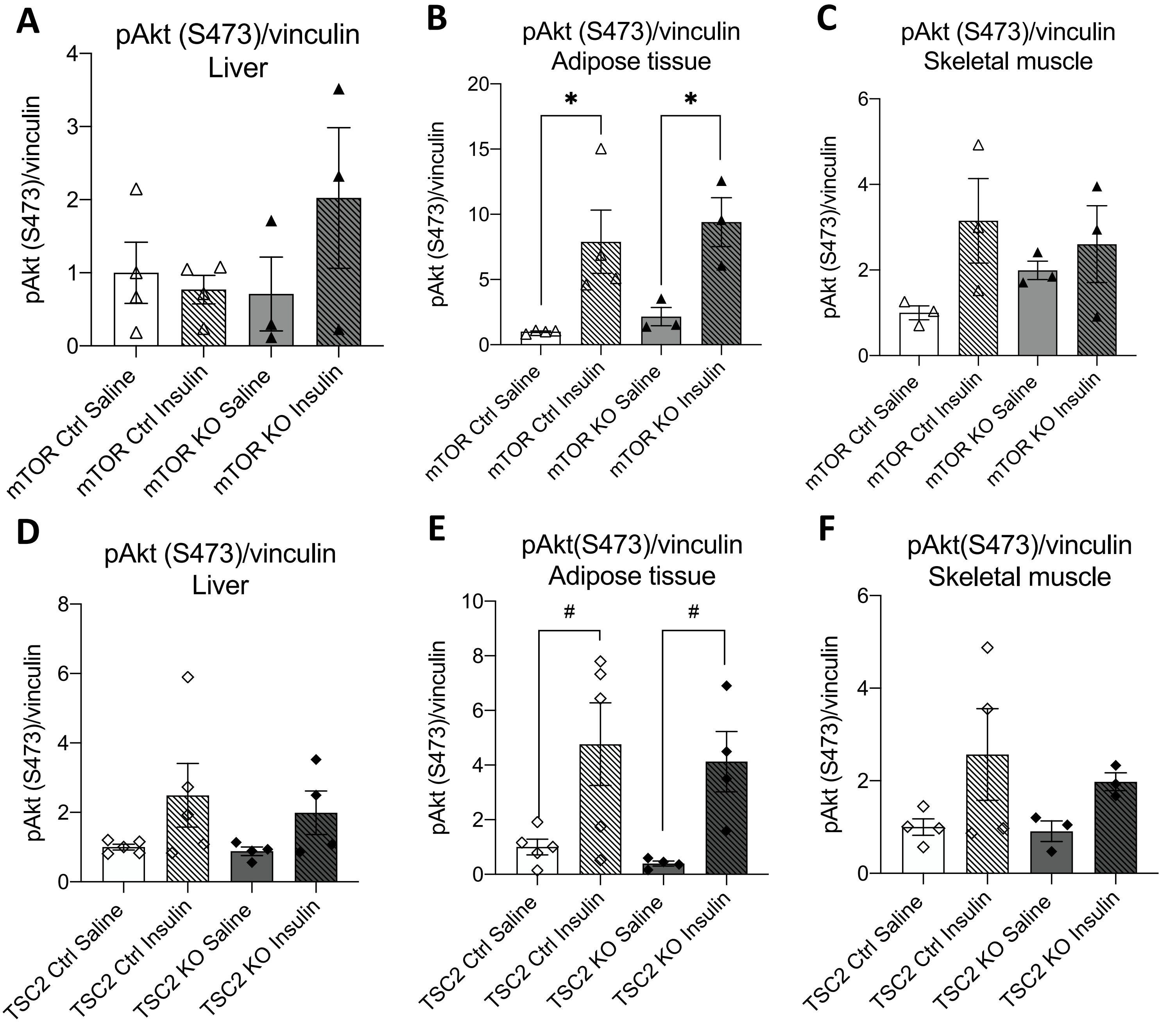

S. Fig. 5. Phospho-Akt levels normalized to vinculin in liver, adipose, and skeletal muscle of placental mTORKO[pl] and TSC2KO[pl] female offspring and littermate controls. Quantification of phospho-Akt normalized to vinculin loading control in (**A**) liver, (**B**) adipose tissue, and (**C**) skeletal muscle of female mTORKO[pl] and littermate controls with and without insulin stimulation (n=3-4). Quantification of phospho-Akt normalized to vinculin loading control in (**D**) liver, (**E**) adipose tissue, and (**F**) skeletal muscle of female TSC2KO[pl] and littermate controls with and without insulin stimulation (n=3-5). Statistical analyses were conducted using one-way ANOVA with Tukey's post-hoc test, with significance *p<0.05, **p<0.01.

A

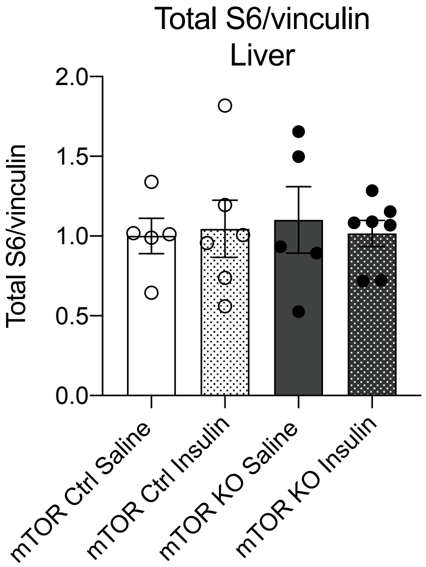

B

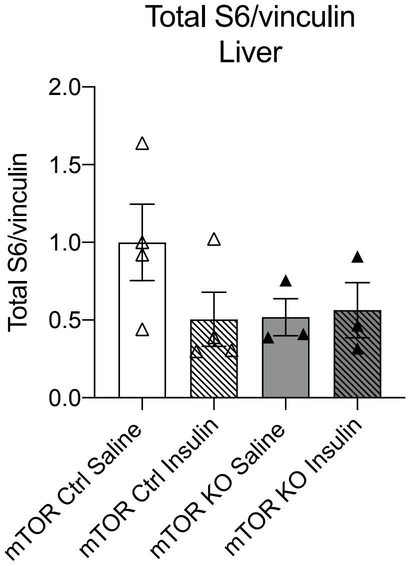

S. Fig. 6. Total hepatic S6 of placental mTORKO^{pl} and littermate controls. Quantification of total S6 normalized to vinculin loading control in liver of (**A**) male mTORKO^{pl} and littermate controls with and without insulin stimulation (n=5-7), and (**B**) female mTORKO^{pl} and littermate controls with and without insulin stimulation (n=3-4). Statistical analyses were conducted using one-way ANOVA with Tukey's post-hoc test, with significance *p<0.05.

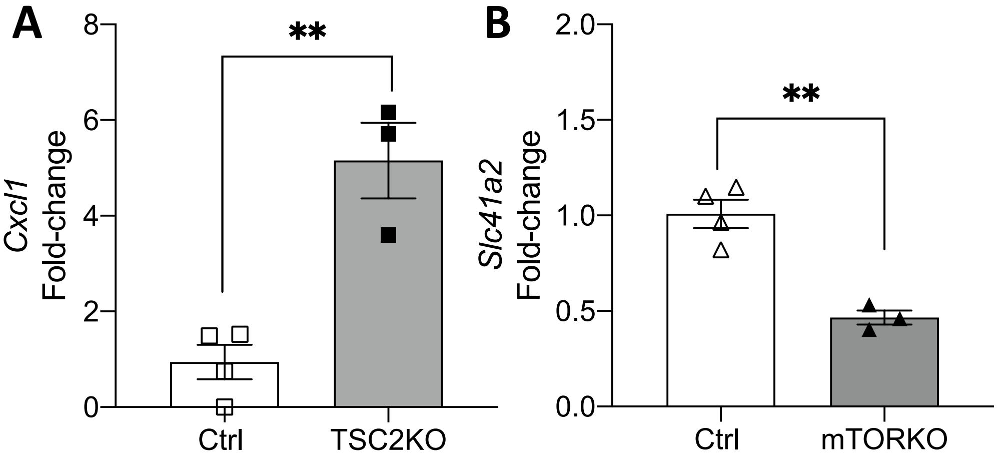

S. Fig. 7. Validation of RNA sequencing candidates. Hepatic (**A**) *Cxcl1* mRNA expression in TSC2KO^{pl} males compared to littermate controls (n=3-4), and (**B**) *Slc41a2* mRNA expression in mTORKO^{pl} females compared to littermate controls (n=3-4). Statistical analyses were conducted using a Student's t-test, with significance *p<0.05.

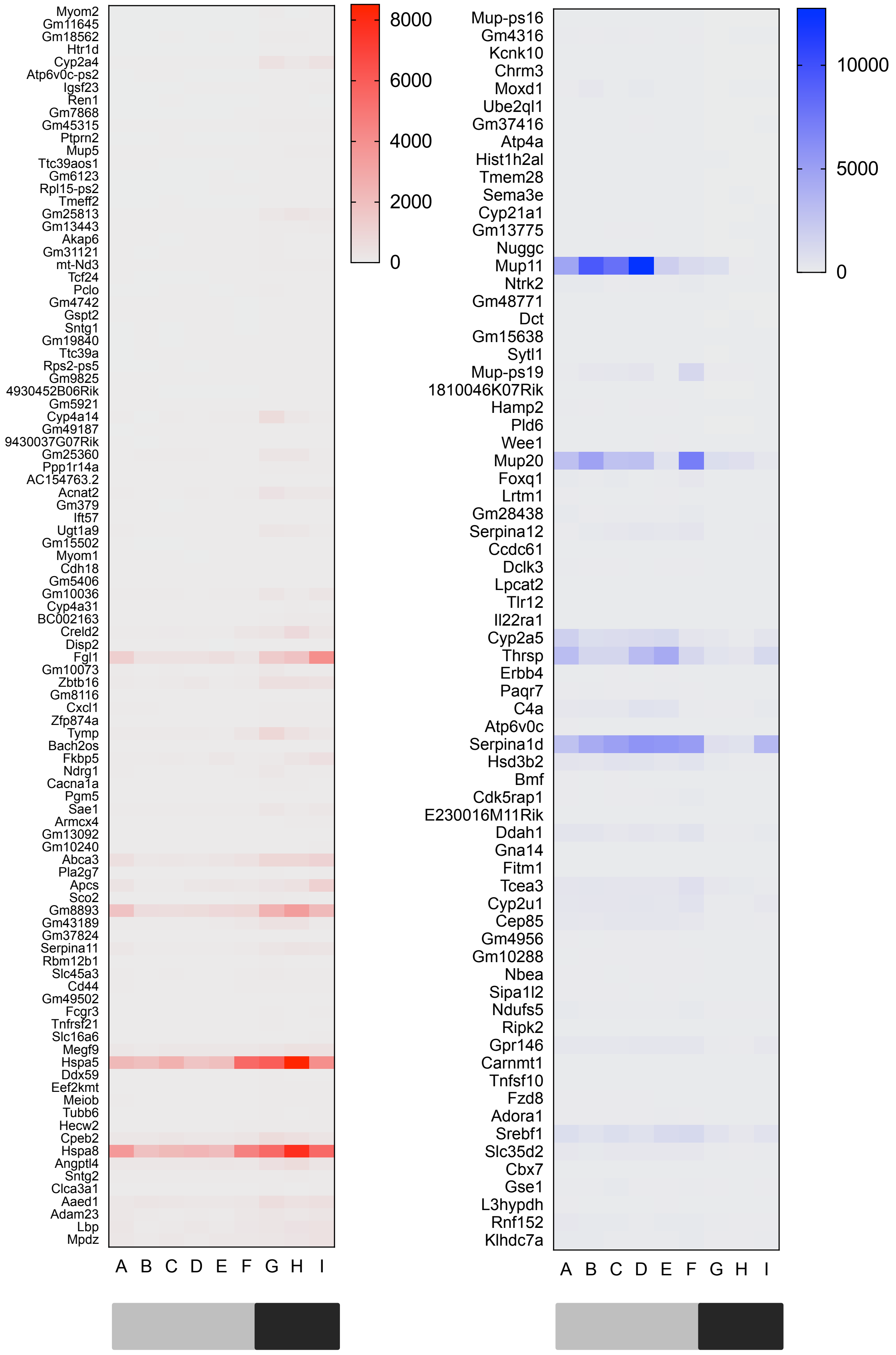

Male liver: mTORKO^{pl} versus TSC2KO^{pl}

S. Fig. 8. RNA sequencing comparisons of male mTORKO^{pl} and TSC2KO^{pl} male offspring. Fold-change>2 and FDR<0.05. Red indicates increased expression. Blue indicates decreased expression.

Figure S9

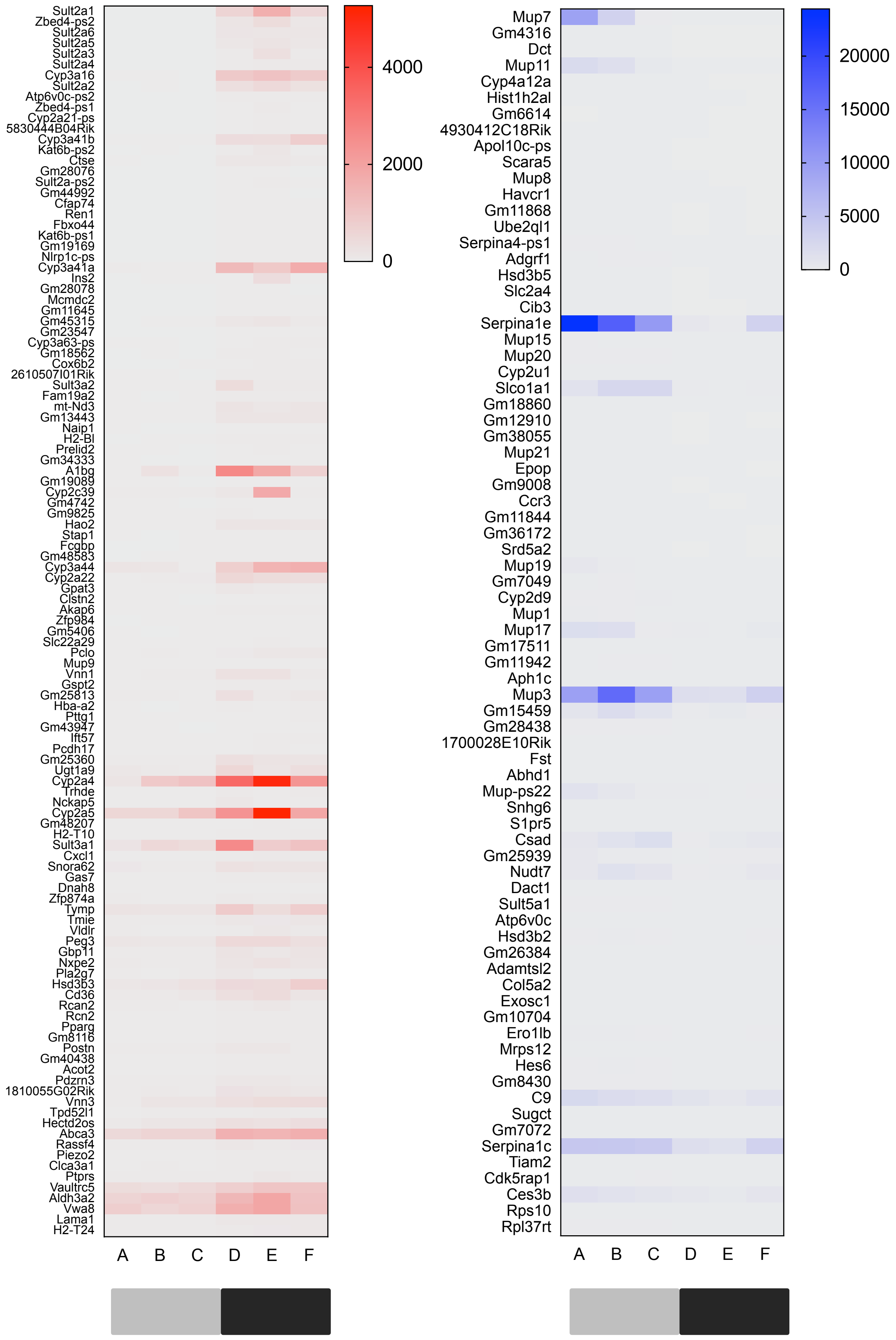

Female liver: mTORKO[pl] versus TSC2KO[pl]

S. Fig. 9. RNA sequencing comparisons of female mTORKO[pl] and TSC2KO[pl] male offspring. Fold-change>2 and FDR<0.05. Red indicates increased expression. Blue indicates decreased expression.

Figure S10

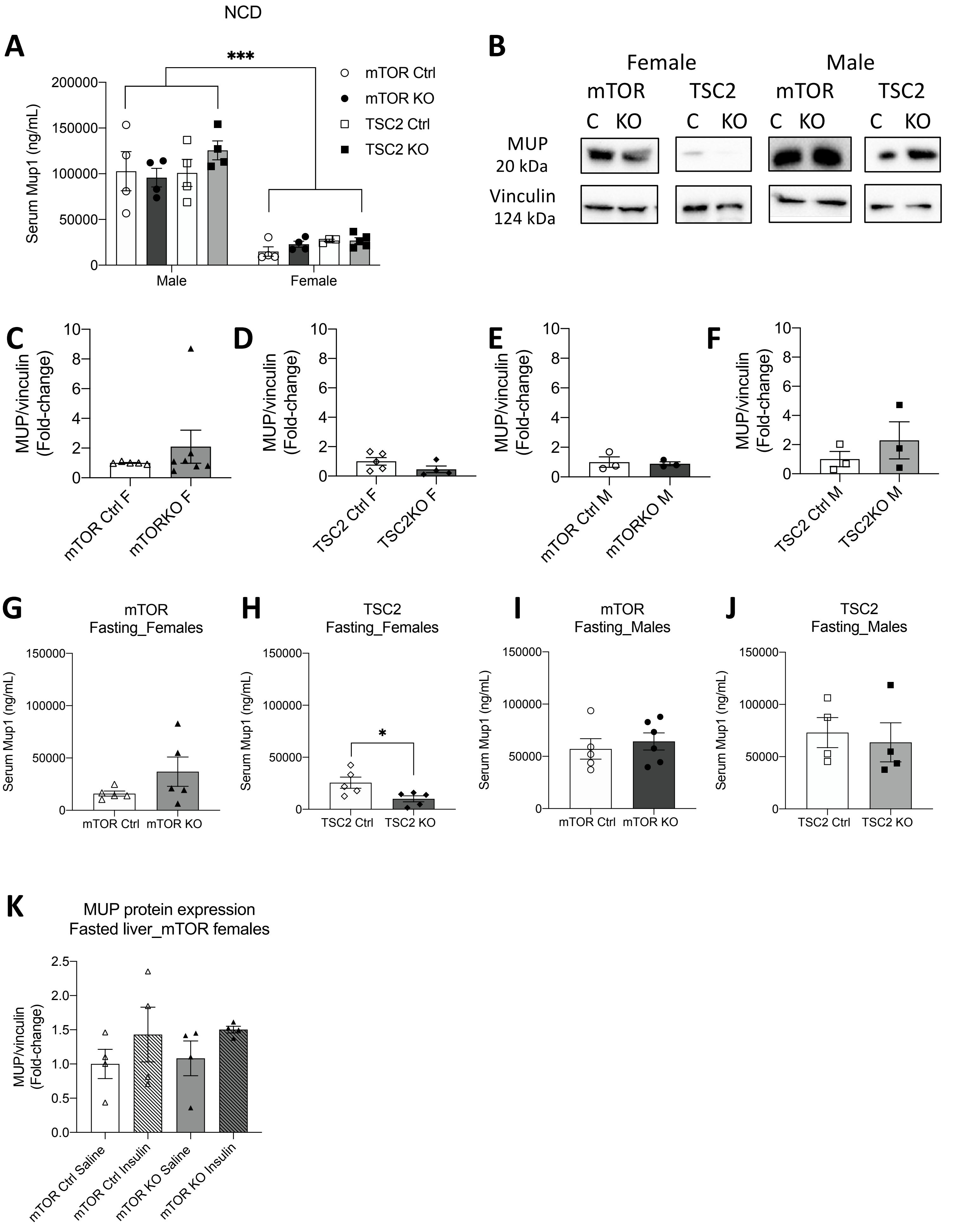
NCD
A
***
mTOR Ctrl
mTOR KO
TSC2 Ctrl
TSC2 KO
Serum Mup1 (ng/mL)
200000
150000
100000
50000
0
Male
Female
B
Female
Male
mTOR
TSC2
mTOR
TSC2
C
KO
C
KO
C
KO
C
KO
MUP
20 kDa
Vinculin
124 kDa
C
D
E
F
MUP/vinculin
(Fold-change)
10
8
6
4
2
0
mTOR Ctrl F
mTORKO F
TSC2 Ctrl F
TSC2KO F
mTOR Ctrl M
mTORKO M
TSC2 Ctrl M
TSC2KO M
G
mTOR
Fasting_Females
H
TSC2
Fasting_Females
I
mTOR
Fasting_Males
J
TSC2
Fasting_Males
Serum Mup1 (ng/mL)
150000
100000
50000
0
*
mTOR Ctrl
mTOR KO
TSC2 Ctrl
TSC2 KO
K
MUP protein expression
Fasted liver_mTOR females
MUP/vinculin
(Fold-change)
2.5
2.0
1.5
1.0
0.5
0.0
mTOR Ctrl Saline
mTOR Ctrl Insulin
mTOR KO Saline
mTOR KO Insulin

S. Fig. 10. Comparing serum and hepatic protein levels of Mup1 between males and females, in fasting state, and in response to insulin. (**A**) Circulating Mup1 levels in female (right) and male (left) mTORKO^{pl} and TSC2KO^{pl} offspring and littermate controls. (**B**) Representative Western blots of MUP and vinculin from livers of 90-day-old female and male offspring; mTORKO^{pl} and littermate control females quantified in (**C**, n=5-7), TSC2KO^{pl} and littermate control females quantified in (**D**, n=4-5), mTORKO^{pl} and littermate control males quantified in (**E**, n=3), and TSC2KO^{pl} and littermate control males quantified in (**F**, n=3). Western blot quantification represented as fold-change relative to littermate controls. Fasting serum levels of Mup1 in (**G**) mTORKO^{pl} and littermate control females (n=5), (**H**) TSC2KO^{pl} and littermate control females (n=5), (**I**) mTORKO^{pl} and littermate control males (n=5-6), and (**J**) TSC2KO^{pl} and littermate control males (n=4). (**K**) Quantification of Western blot measuring hepatic MUP expression in mTORKO^{pl} females and littermate controls treated with 1U/kg insulin or control-treated with saline. Statistical analyses were conducted using an unpaired two-tailed t-test or a one-way ANOVA with Tukey's post-hoc test, with significance *p<0.05, ***<0.001.

Supplementary Table 1: Gene ontology and IPA results

Gene ontology – Female liver – mTORKO^{pl} versus TSC2KO^{pl}	Count	p-value
Oxidation-reduction process	25	6.2E-7
Lipid metabolic process	28	6.2E-7
Negative regulation of insulin secretion	6	4.9E-5
Mitochondrion morphogenesis	6	1.4E-4
Glucose homeostasis	11	2.2E-4
Negative regulation of peptide secretion	7	3.7E-4

Gene ontology – Male liver – mTORKO^{pl} versus TSC2KO^{pl}	Count	p-value
Lipid metabolic process	20	1.7E-1
Signal release	11	1.7E-1
Regulation of hormone levels	11	2.3E-1
Positive regulation of glucose metabolic process	4	3.7E-1
Neurotransmitter transport	6	3.7E-1
Cell-cell signaling	17	3.7E-1

IPA – Female liver – mTORKO^{pl} versus TSC2KO^{pl}	Count	p-value range
Endocrine System Disorders	26	9.13E-3-1.50E-5
Metabolic Disease	31	9.13E-3-1.50E-5
Organismal Injury and Abnormalities	85	9.13E-3-1.50E-5
Gastrointestinal Disease	67	9.13E-3-8.65E-5
Renal and Urological Disease	18	9.13E-3-8.65E-5

IPA – Male liver – mTORKO^{pl} versus TSC2KO^{pl}	Count	p-value range
Cancer	107	5.06E-3-2.07E-6
Hematological Disease	53	5.06E-3-2.07E-6
Organismal Injury and Abnormalities	111	5.06E-3-2.07E-6
Immulogical Disease	55	5.06E-3-5.14E-6
Gastrointestinal Disease	98	5.06E-3-6.93E-6

Supplementary Table 2: RT-qPCR primer sequences

Gene	Forward Primer	Reverse Primer
Actin	GCCCTGAGGCTCTTTTCCAG	TGCCACACGATTCCATACCC
Mup1	GAAGCTAGTTCTACGGGAAGGA	AGGCCAGGATAATAGTATGCCA
Cxcl1	AGCCACACTCAAGAATGGTC	GTCTGTCTTCTTTCTCCGTTACTT
Slc41a2	ACAGCAACGCTTGTTCAAATTA	TTCCTCTCTTAAACGTCTGGATTT
